# Neurotransmitter-mediated activity spatially controls neuronal migration in the zebrafish cerebellum

**DOI:** 10.1371/journal.pbio.2002226

**Published:** 2018-01-04

**Authors:** Ulrike Theisen, Christian Hennig, Tobias Ring, Ralf Schnabel, Reinhard W. Köster

**Affiliations:** 1 Technische Universität Braunschweig, Zoological Institute, Cellular and Molecular Neurobiology, Braunschweig, Germany; 2 Technische Universität Braunschweig, Institute for Genetics, Braunschweig, Germany; 3 Technische Universität Braunschweig, Institute for Engineering Design, Vibroacoustics, Braunschweig, Germany; The Salk Institute for Biological Studies, United States of America

## Abstract

Neuronal migration during embryonic development contributes to functional brain circuitry. Many neurons migrate in morphologically distinct stages that coincide with differentiation, requiring tight spatial regulation. It had been proposed that neurotransmitter-mediated activity could exert this control. Here, we demonstrate that intracellular calcium transients occur in cerebellar neurons of zebrafish embryos during migration. We show that depolarization increases and hyperpolarization reduces the speed of tegmental hindbrain neurons using optogenetic tools and advanced track analysis optimized for in vivo migration. Finally, we introduce a compound screening assay to identify acetylcholine (ACh), glutamate, and glycine as regulators of migration, which act regionally along the neurons’ route. We summarize our findings in a model describing how different neurotransmitters spatially interact to control neuronal migration. The high evolutionary conservation of the cerebellum and hindbrain makes it likely that polarization state-driven motility constitutes an important principle in building a functional brain.

## Author summary

Postmitotic neurons migrate from their site of origin to their final destination in the developing brain to form functional structures. These neurons typically follow defined routes through the tissue. Previous studies investigating progress along such route have identified neurotransmitters—chemicals that transmit the signals between neurons—as important regulators in neuronal migration using mostly rodent brain slice cultures and cultivated neurons. In this study, we use live zebrafish embryos to test the influence of neurotransmitters on migrating hindbrain neurons. First, we demonstrate that calcium transients can be measured in these neurons using genetically encoded reporters. Next, we use optogenetic channels to specifically de- or hyperpolarize the plasma membrane of the neurons to show that the polarization state is linked to migratory speed. Finally, we use a screening method to identify the neurotransmitter systems involved in migration progress control. We summarize these findings in a model that suggests that there are regions of influence for different neurotransmitters that act successively on the neurons to ensure their timely arrival at their destination.

## Introduction

The brain is the most complex organ in the body. It carries out its tasks by networks of neurons that form separate spatial entities, which are often organized into layers or nuclei. During embryonic development, many neurons that arise in various progenitor zones migrate in order to build these intricate structures [1]. It is thought that active cell migration is also essential to establish functional networks as neurons encounter their interacting partners along the way [2], and axonogenesis and maturation often coincide with migration [3–5]. Thereby, neuronal migration underlies the formation of functional circuitry [6].

Neuronal migration has been studied in various contexts using mostly in vitro assays or in situ rodent brain slices [7–9]. These studies have identified a number of molecules involved in cell guidance [10], but the control of forward movement is less understood. Still, these studies have discovered that activity mediated by different neurotransmitters has a complex role in the migration of neurons [11]. The best studied neurotransmitter system in this context is glutamate [12], which typically promotes migration [13–16]. The function of other neurotransmitters, such as acetylcholine (ACh), glycine, and GABA is less clear, but may depend on the type of neuron studied and assay conditions [17–24]. Studies using implants in rodent brains have been able to show that glutamate is a major influence in the correct arrangement of neurons into layers during development [25], yet this sophisticated technology did not allow for the live monitoring of cell migration. It has been proposed that different neurotransmitters act together or sequentially to fine-tune migration [12,26,27], but in vivo data from neurons migrating in their natural environment without incurring tissue damage from invasive procedures are scarce [25,28].

Here, we use live zebrafish embryos to track individual neurons along their route in the developing cerebellum to investigate neurotransmitter-mediated membrane polarization in neuronal migration. In the adult zebrafish brain, tegmental hindbrain nuclei neurons (THNs) form clusters that are involved in gustatory, viscero-sensory, and visual information processing [29]. These are homologous to the mammalian parabrachial, parabigeminal, and laterodorsal-pedunculopontine tegmental hindbrain nuclei. During embryonic development, THNs arise at the upper rhombic lip (URL) of the cerebellar primordium between 24 hours post fertilization (hpf) and 48 hpf [29,30]. They emigrate from the URL in a first phase to reach the midbrain-hindbrain boundary (MHB) [30]. THNs then follow the MHB in a second migratory phase to reach their destination at the ventral end of the MHB [4,30]. This migration in distinct phases suggests that the necessary spatiotemporal control could be exerted by neurotransmitter-mediated activity as a fast acting, spatially limitable regulator.

Zebrafish embryos rapidly develop their most important brain regions while they are still translucent. This makes them an ideal model for tracking individual cells in their natural environment without damaging the surrounding tissue. Early zebrafish embryos are also easily accessible for pharmacological intervention and optogenetic manipulations by light microscopy. Taking advantage of these properties, we demonstrate that calcium transients are present in the THNs, and that de- and hyperpolarization produce opposite effects on THN migration speed in spatially defined regions. We identify ACh, glycine, and glutamate as key neurotransmitters in the regulation of migration. These findings are summarized in a model outlining the interactions of the neurotransmitters to spatially control THN migration.

## Results

### THNs move along a defined route and change their morphology along the way

**Fig 1:**
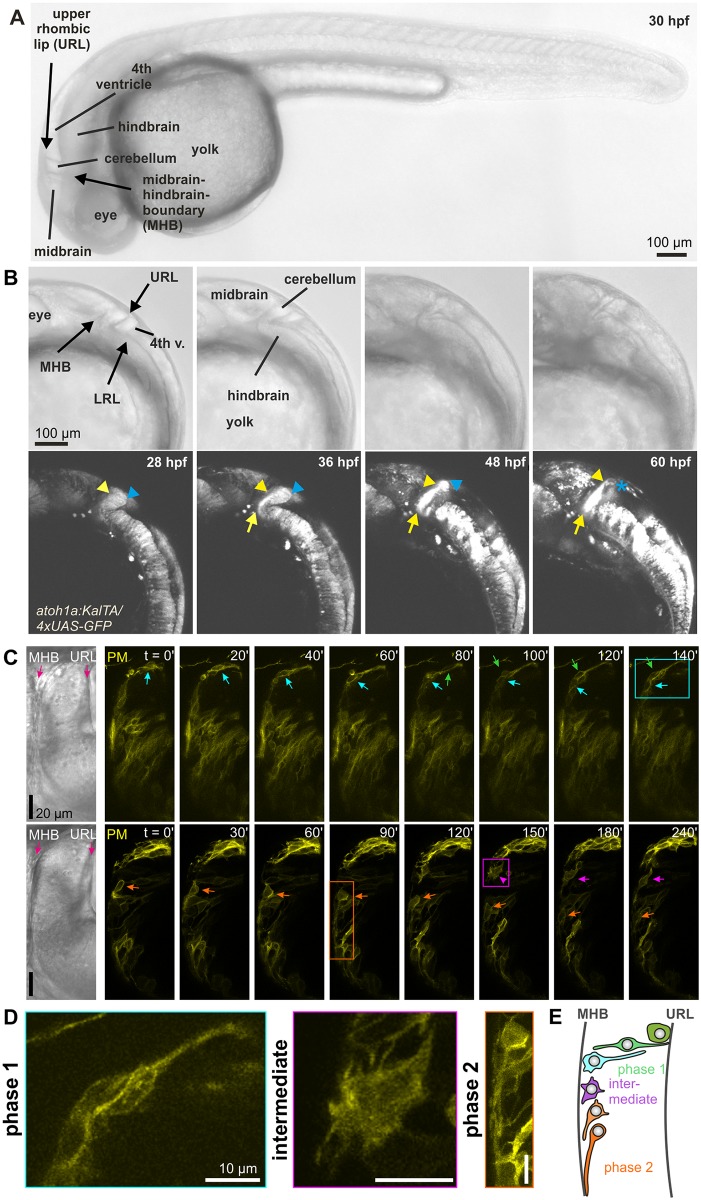
THNs migratory route and associated morphological changes. (A) Anatomical overview of *atoh1a*:*KalTA/4xUAS-GFP*-expressing larvae from 30 hpf. Prominent features are indicated in transmitted-light images (top panel). Scale bar: 100 μm. For further details about THN position in adults, refer to [29]. (B) THNs are identified in the lower panel by their fluorescence and position in the cerebellum between URL and MHB, as well as their expression of GFP from an *atoh1a* promoter. Over time, cells migrate towards the MHB, which they follow ventrally to form a large cluster. Blue arrowheads and stars indicate start position, yellow arrowheads migrating THNs, yellow arrows point to the emerging THN cluster at the ventral end of the MHB. Anatomical features are indicated in the transmitted light images in the top row. Scale bar: 100 μm. See also S1 Video. (C) Morphologically, THNs begin their migration as very elongated cells that remain in contact with the URL until the main cell body has reached the MHB (arrows; top panel). At the MHB, dorsal cells exhibit a poorly polarized stage with many protrusions (arrowhead). Cells positioned more ventrally at the MHB assume a unipolar morphology with the protrusion extending ventrally (arrows). THNs transiently express PM-targeted YFP. Elapsed time is indicated at the top. Scale bar: 20 μm. Colored frames indicate the cells shown in (C). See also S2 Video. (D) Higher magnification of THNs indicated in (B), illustrating the morphological changes of THNs along their migratory route. Scale bar: 10 μm. (E) Schematic representation of THN morphology in different phases of migration. *atoh1a*; *atonal 1a*; GFP, green fluorescent protein; hpf, hours post fertilization; LRL, lower rhombic lip; MHB, midbrain-hindbrain boundary; PM, plasma membrane; THNs, tegmental hindbrain nuclei neurons; UAS, upstream activating sequence; URL, upper rhombic lip; YFP, yellow fluorescent protein.

The URL in zebrafish embryos is a germinal zone along the dorsoposterior surface of the developing cerebellum, which gives rise to different long-distance migrating neurons, such as THNs and cerebellar granule cells, similar to the situation in mammals [29]. THNs are first to emerge from the URL from 24 hpf and traverse the whole cerebellar primordium to reach the tegmental hindbrain. Early emerging neurons, THNs are characterized by the expression of the transcription factor *atonal 1a (atoh1a)* [4]. Long-term in vivo imaging of THNs expressing green fluorescent protein (GFP) in a bipartite Gal4 (transcriptional activator protein)/upstream activating sequence (UAS) system driven by the *atoh1a* promoter [4] shows that THNs first reach the MHB after leaving the URL (Fig 1A and 1B). Next, they follow the MHB ventrally, where they form clusters in the tegmentum of the hindbrain from around 60 hpf onwards (S1 Video). These separate into the secondary gustatory/viscerosensory nucleus, the nucleus isthmi, and the superior reticular nucleus, which contain cholinergic neurons during further development, and establish connections in the di- and mesencephalon similar to the equivalent nuclei in mammals [29].

Along this migratory route, THNs change shape dramatically. In the first migratory phase between URL and MHB, THNs exhibit an elongated bipolar shape and retain contact with the URL at the rear (Fig 1C, top panel, and zoom in left in Fig 1D). Once the nucleus is located near the MHB, THNs retract their trailing ends. THNs that reach the MHB at its dorsal end often exhibit a brief, highly protrusive phase. These short-lived protrusions extend in all directions (Fig 1C, bottom panel, and middle image in Fig 1D). In contrast, THNs that reach the MHB more ventrally often directly assume the morphology of migratory phase 2, which consists of a very elongated leading process with the nucleus situated at the rear (Fig 1C, bottom panel and Fig 1D, right; see also S2 Video). A schematic overview of THN morphology along their migratory route is depicted in Fig 1E. These changes suggest a close co-regulation of the morphology and migratory progress associated with the specific region that the cells migrate through. Results from rodent neurons tracked in in situ brain slices [24,31] suggest that locally regulated, neurotransmitter-mediated plasma membrane (PM) potential changes could exert this control on THN migration in vivo.

### Calcium signals arise in THNs along their migration route

**Fig 2:**
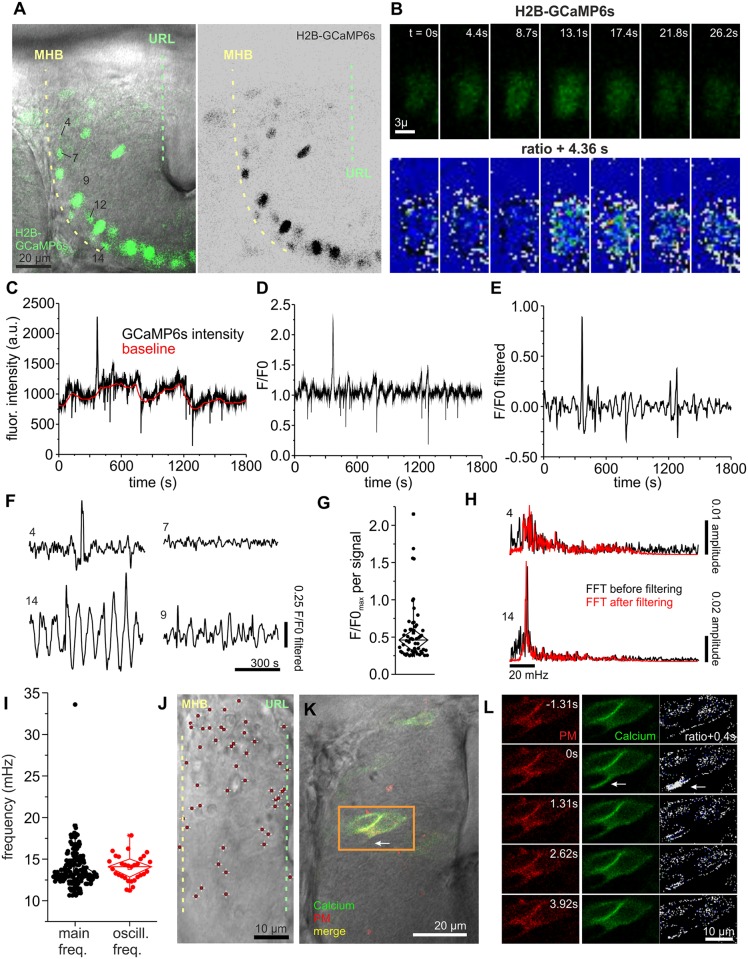
Calcium signals indicate the presence of activity in the cerebellum. (A) As THNs differentiate along route, only THNs in phase 2 show expression of H2B-GCaMP6s from the *elavl3* promoter, which increases in strength towards the ventral end of the MHB. Numbers identify the nuclei shown as examples in this figure. Scale bar: 20 μm. (B) Stills from the fluorescence recording of nucleus 12 shown in (B), top panel, demonstrate the presence of calcium transients. In the lower panel, a ratiometric image comparing subsequent frames of the sequence in the top panel is given. White indicates high-intensity divergence and blue indicates low-intensity divergence. Scale bar: 3 μm. (C) Fluorescence intensity measurement F of nucleus 12 for the whole observation time is shown in black and the calculated local baseline F0 in red. (D) Converting these into F/F0 shows that nucleus 12 exhibited only one prominent calcium event with smaller amplitudes difficult to judge due to the presence of noise. (E) After application of a bandpass filter, noise and false-positive signals are removed from the trace, whereas actual signals become more prominent. (F) Different patterns of calcium transients are shown for several nuclei imaged simultaneously. Nucleus 4 exhibits a single event like nucleus 12, whereas nucleus 7 is silent during the observation time. Nucleus 14 represents very regular calcium oscillations that were observed in a fraction of nuclei, whereas nucleus 9 appears to be a mixture of several types. (G) Plotting maximal amplitude values for each signal demonstrates that a wide range of signal strengths can be detected in THNs. (H) Fourier transformation analysis of the various traces illustrates that some nuclei have very regular signals at a distinct frequency (bottom), whereas others with a low incidence of calcium events do not show this preference (top). For control, results were compared for filtered (red) and unfiltered (black) traces. (I) All 195 FFTs were analyzed for the most prominent frequency by plotting the frequency corresponding to the maximal amplitude value. Although this represents the range of frequencies contained in the dataset, the frequency of regular oscillations can be obscured by the inclusion of other signal patterns. Hence, 32 nuclei with strong regular calcium oscillations were selected and their oscillation frequency determined by Gaussian fits. The frequencies of these oscillations are centered around a mean value of 14.1 mHz. (J) Calcium signals are detected in THNs throughout the cerebellum. The positions of THNs transiently expressing PM-bound GCaMP6s and exhibiting at least one signal during the observation period of 30 min were superimposed to create a map (*N* = 50). Scale bar: 10 μm. (K) Example of a THN exhibiting localized calcium signals, showing the position of the cell in the tissue. Scale bar: 20 μm. (L) Stills from the recording of THNs indicated in (K). Ratiometric images comparing the fluorescence of GCaMP6s in subsequent stills relative to the frame with the highest signal are shown (white indicates high, blue low signal difference). Arrow points to the calcium signal. Elapsed time relative to the frame with the highest GCaMP6s signal is indicated on the images. Scale bar: 10 μm. See also S3 Video. Data depicted in the graphs can be accessed in S1 Data. F/F0, fluorescence over background; FFT, fast Fourier transform; flour, fluorescent; freq, frequency; GCaMP6, genetically encoded calcium sensor, circular permutated green florescent protein-Calmodulin-M13 peptide 6; H2B, Histone 2B; MHB, midbrain-hindbrain boundary; oscill, oscillation; PM, plasma membrane; THN, tegmental hindbrain nuclei neuron, URL, upper rhombic lip.

Neurotransmitters evoke intracellular calcium transients as propagating signals (for review see [11]). This prompted us to determine if calcium transients can be detected in THNs. In order to improve the quantification of calcium transients, we made use of the *Tg(elavl3*:*Hsa*.*H2B-GCaMP6s)* fish line (kindly provided by Misha Ahrens, [32]). In these embryos, the nuclear localized calcium sensor is easy to track and measure in all mature neurons. Out of all Histone 2B (H2B) fused to genetically encoded calcium sensor, circular permutated green florescent protein-Calmodulin-M13 peptide 6s (GCaMP6s)-expressing neurons, we selected those as THNs that (1) were positioned at the MHB at several μm depth from the body surface, (2) were oriented towards the ventral end of the MHB based on the long axis of the elliptic nuclei, and (3) expressed the marker at increasing strength towards the ventral end of the MHB, in line with the observation that THNs begin their differentiation along route at the MHB [4] (Fig 2A). The GCaMP6s fluorescence of these nuclei was recorded in 30-min videos at 436 ms intervals in a single z plane. We measured the total fluorescence intensity of individual nuclei by region of interests (ROIs) that the nucleus under investigation occupied during the observation window. Due to tissue shifts arising from embryo growth, active migration and pressure from the surrounding cells, some nuclei left the focal plane or were joined by other nuclei that invaded the ROI. In these cases, we included only data from the durations in which the nuclei remained alone and in focus in their ROIs in our analysis (Fig 2B for example stills from nucleus 12 indicated in 2A). These raw traces were background-corrected by calculating the baseline intensity value (F0) for each timepoint from all values in a 100 frame window ([33]; Fig 2C for example nucleus 12). Dividing the raw fluorescence intensity (F) by this baseline value at each timepoint produced the fluorescence over background (F/F0) trace depicted in Fig 2D. The resulting traces were still rather noisy, especially in nuclei with low expression levels of H2B-GCaMP6s. The noise was removed by applying a bandpass filter with cutoff frequencies at 20 and 200 mHz, respectively (Fig 2E). A calcium transient was detected when the amplitude of this F/F0 trace after filtering reached >0.25, and was checked against the original film to remove false-positives.

Using this method, we analyzed 195 nuclei from 15 embryos and detected a total of 55 calcium transients. We noticed that different patterns of calcium transients occur in THNs (Fig 2F); many nuclei showed mostly single short-term peaks, while others appeared dormant (no signal). The strengths of individual signals as seen in the maximal values for each signal in the F/F0 trace varied widely (Fig 2G, signals pooled from all 195 nuclei; S1 Data). We also noticed that a significant portion of nuclei exhibited very regular calcium transients that occurred at low F/F0 amplitudes, and were therefore excluded from the previous analysis by setting the 0.25 cutoff level (trace 14 in Fig 2F). To determine the frequency spectrum contained in the data, all F/F0 traces were Fourier transformed (Fig 2H; for control, the transformations were repeated with unfiltered datasets to avoid losing frequency information). From these fast Fourier transform (FFT) values, we first determined the dominant frequencies contained in each trace by identifying the frequencies with the highest amplitude in each FFT (Fig 2I, S1 Data). These spread over a range of frequencies when looking at all 195 nuclei. As the full dataset includes nuclei without prominent oscillations, which could mask results for the oscillating population, we selected 32 nuclei out of the 195 total number, which showed very regular oscillations by visual inspection of the F/F0 traces, and calculated their mean oscillation frequency by Gaussian fitting to the FFTs. This yielded an oscillation frequency close to 14 mHz (Fig 2I, S1 Data). This value is close to the 16.7 mHz used in the optogenetics experiments (see next paragraph). Hence, we tested all 195 FFTs for the prominence of 16.7 mHz ± 1.67 mHz. Surprisingly, this analysis revealed that signal components in this range occur in approximately 90% of all nuclei (*N* = 176). Yet in only 32.3% of all nuclei (*N* = 63), this frequency is prominent, or corresponding to the highest amplitude of the FFT within the 16.7 mHz ± 1.67 mHz frequency range.

Taken together, it appears likely that a mix of stronger, individual calcium events and low-level oscillations exists in many THNs. A further investigation of the types of calcium signals in THNs, as well as the relevance of individual calcium transient types for THN migration, will continue in the future as better calcium signal detection technology for THNs becomes available.

Although these results clearly show that calcium transients occur in THNs at the MHB, the limitations set by the confinement of H2B-GCaMP6s expression in THNs to phase 2 THNs at the MHB does not allow us to infer the extent of calcium transients occurring along the whole migration route from the URL. Likewise, we could miss hotspots of activity that occur in the cerebellum. Consequently, we co-expressed a PM-targeted version of GCamp6s as calcium sensor and FyntagRFP-T for positional control from a bidirectional vector in THNs, taking advantage of the Gal4/UAS system driven by the *atoh1a*-promoter [4]. THNs were again continuously imaged for 30 min in a single focal plane. Next, THNs were screened for calcium transients, which were defined by an increase of green fluorescence of at least 2-fold over baseline, whereas the red fluorescence remained unchanged (S1 Fig). Next, the position of every signaling THN was marked within the cerebellum using the transmitted-light channel images. These images were registered using the dorsal surface of the cerebellum and the MHB as anatomical landmarks, and then individually size-adjusted to align at their URLs. All signaling cell positions (*N* = 50 cells out of a total of 238 analyzed from 20 embryos) were superimposed to produce the image in Fig 2J (see also S1 Fig), illustrating that calcium transients of widely varying duration and rate can be recorded throughout the tissue (see S1 Fig). Taking a closer look at these signals, we observed that the calcium transients tend to occur at the front of the THN in about one-third of the cells (Fig 2K and 2L; see also S1 Fig and S3 Video). This phenomenon has been described before in cells migrating in vitro and slice culture [34] [35].

In sum, these results demonstrate that calcium transients occur in many THNs, with a tendency to arise at the front of migrating THNs. The calcium transients exhibit diverse patterns and occur throughout the migratory route of THNs in the cerebellum. Such calcium transients could be caused by neurotransmitter-induced changes of the polarization state of the neurons [7].

### Hyperpolarization decreases and depolarization increases THN migration

The presence of calcium transients at the time of THN migration could be due to neuronal activity. Therefore, we directly manipulated activity in THNs to analyze the effects on migration by cell tracking using optogenetics to circumvent the need to manipulate specific neurotransmitters at this point of the investigation.

**Fig 3:**
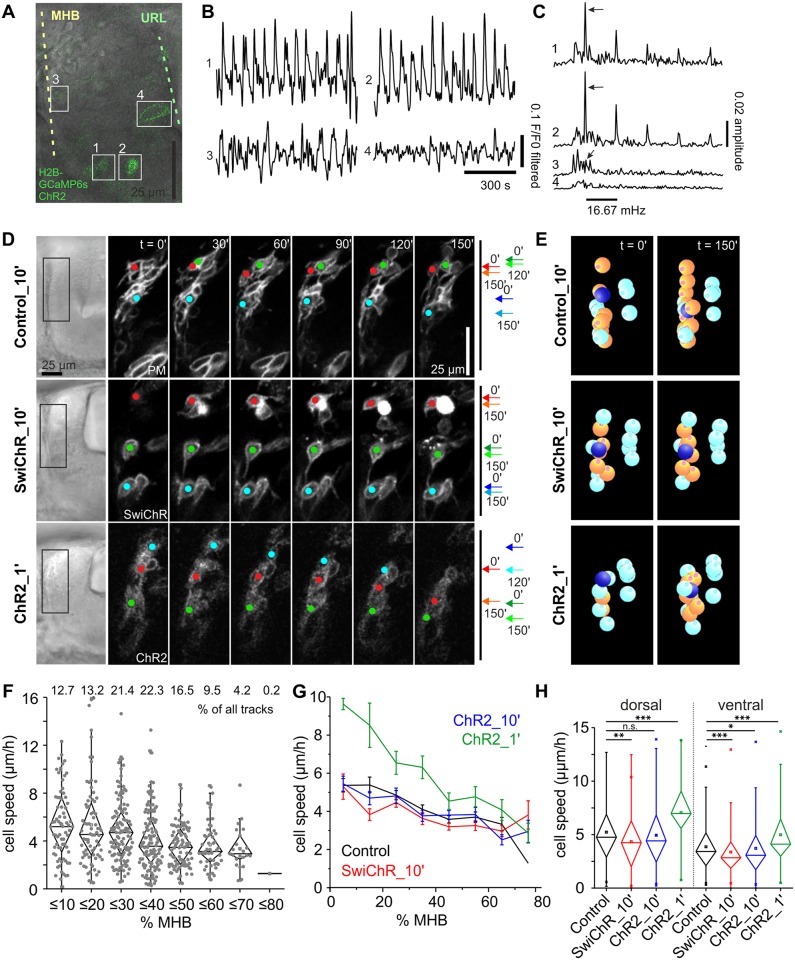
Optogenetic activity control influences THN migration. (A) Overlay of fluorescent and transmitted-light images of a 30 hpf embryo co-expressing H2B-GCaMP6s and ChR2-YFP. At this developmental stage, only nuclei at the ventral end of the MHB express H2B-GCaMP6s at high enough levels to detect fluorescence signal increases over the background fluorescence from ChR2-YFP (nuclei 1 and 2, compare to nucleus 3 in the central region of the MHB). Nuclei at the URL, however, express only ChR2-YFP and serve as negative control (nucleus 4). Scale bar: 25 μm. (B) Nuclei co-expressing both markers show highly regular calcium transients (nuclei 1 and 2), while negative control THNs do not (nuclei 3 and 4). (C) Fourier transformation reveals that the frequency of calcium transients in nuclei 1 and 2 strongly coincides with the expected frequency of one signal per min, or 16.7 mHz (arrows). Nucleus 3 shows a weak peak at this frequency. (D) Tracking of THNs expressing either control (YFP-CAAX), ChR2-YFP, or SwiChR-YFP along the MHB (colored dots) demonstrates that hyperpolarization slows THNs down, while depolarization increases speed. Transmitted light images on the left provide positional information on THNs within the tissue; boxes indicate the region shown at greater magnification in the fluorescence images. Migration progress over time is schematically indicated on the right, in which arrows indicate the start and end points for the shown frames. Elapsed time is indicated at the top. Scale bar: 25 μm. See also S4 Video. (E) Individual THNs (orange) were tracked in SIMI°BioCell software for quantification. An example is marked in dark blue. The tracks were corrected for tissue shifts using reference points (cyan). See also S5 Video. (F) THNs reduce their migration speed as they progress along the MHB from approximately 25% to −35% of the distance to the ventral end. Zero percent is the dorsal and 100% is the ventral end of the MHB. Track distribution is given at the top. (G) Migration speeds are affected by de- and hyperpolarization irrespective of starting point. Means ± SEM are plotted per 10% MHB bin. (H) Tracks were grouped as either dorsal (≤ −27.5% MHB) or ventral (≥27.5% MHB) to test the significance of de- and hyperpolarization compared to control level. Only significant differences are indicated. Data depicted in the graphs can be accessed in S2 Data. CAAX, PM-targeting signal derived from K-Ras; ChR2, channelrhodopsin; F/F0, fluorescence over background; GCaMP6, circular permutated green florescent protein-Calmodulin-M13 peptide 6s; H2B, Histone 2B; hpf, hours post fertilization; MHB, midbrain-hindbrain boundary; n.s., not significant; PM, plasma membrane; SwiChR, mutated channelrhodopsin; THN, tegmental hindbrain nuclei neuron; URL, upper rhombic lip; YFP, yellow fluorescent protein.

As the morphological changes in THNs are most prominent at the MHB, we focused our analysis on this region. To test the suitability of the optogenetic channels for inducing changes to the calcium transients in THNs, we first expressed the channels using cytomegalovirus (CMV) promoters in *Tg(elavl3*:*Hsa*.*H2B-GCaMP6s)* embryos (Fig 3A and S2 Fig). We expressed either channelrhodopsin (ChR2) or mutated channelrhodopsin (SwiChR), each tagged with YFP for identification. ChR2 is a commonly used cation channel that depolarizes cells upon application of blue light. SwiChR is a mutated version of ChR2 that retains the same activation spectrum but allows the inward passage of anions, thereby hyperpolarizing cells [36]. The fact that ChR2 and SwiChR share the same physical and biological parameters, e.g., ion influx rates, expression levels of the proteins and protein architecture, facilitates the comparison between de- and hyperpolarization effects.

We depolarized cells by blue light illumination for a duration of 6.57 μs/pixel using a confocal microscope. This is equivalent to the exposure to the blue light for a total duration of 1.64 ms every 60 s for an average sized THN. The fluorescence of the calcium sensor was measured in cells located at the ventral end of the MHB, as these expressed the H2B-GCaMP6s most strongly (nuclei 1 and 2 in Fig 3A–3C). Regular increases in fluorescence intensity (Fig 3B) occurred at a frequency of 16.7 mHz, as is expected if ChR2 depolarizes cells in response to a blue light pulse every 60 s (arrows in Fig 3C). All 20 nuclei from eight embryos that were analyzed in this way showed the same response at 16.7 mHz. Thirty low-expressing cells more dorsal at the MHB or cells at the URL that do not express H2B-GCaMP6s served as internal negative controls (nuclei 3 and 4 in Fig 3B and 3C). Sixteen of these control nuclei from 11 embryos showed a weak response at 16.7 mHz in this analysis, likely due to the weaker expression of GCaMP6s relative to ChR2-YFP. Fourteen nuclei did not show any frequency preference (nucleus 4 in Fig 3C). As additional controls, we repeated the experiment in SwiChR-YFP expressing cells in the same genetic background, as well as in *atoh1a*:*KalTA*-positive embryos co-expressing a nuclear localized GCaMP6s and either ChR2-YFP or SwiChR-YFP. In these experiments, neither of the SwiChR-expressing cells showed a calcium signal upon blue light illumination, whereas ChR2-positive cells responded as expected to the channel opening at 16.7 mHz (S2 Fig).

Having established that the optogenetic tools allow us to de- and hyperpolarize cells migrating in the cerebellum, we stimulated *atoh1a*:*KalTA*-positive embryos expressing either ChR2-YFP or SwiChR-YFP in THNs every 10 min for 4 h with 476 nm illumination at 14.77 μs/pix or 3.15 ms for an average sized THN to open the respective channel, and 514 nm illumination to locate the THNs (Fig 3D). In addition, we used PM-targeted YFP as migration control for these illumination settings (see also S4 Video). To determine migration speeds of individual THNs, the cells were tracked in SIMI°BioCell [37] (orange spheres in Fig 3E, see also S5 Video), and the tracks were subsequently corrected for tissue shift (reference markers as cyan spheres in Fig 3E; *N* [control] = 569 tracks/23 embryos, *N* [ChR2_10ʹ] = 557 tracks/33 embryos, *N* (ChR2_1ʹ) = 175 tracks/15 embryos, *N* [SwiChR_10ʹ] = 594 tracks/32 embryos; see also S1 Table for *N* and statistic parameters). Next, tracks were classified by their starting point in bins of 10% of total MHB length, where 0% is the dorsal and 100% the ventral end. This analysis revealed that THNs begin to slow in their progression along the MHB from approximately 25%–35% MHB on (Fig 3F and 3G; S2 Data). As this speed profile suggested that dorsal tracks are different from ventral tracks, we considered dorsal and ventral tracks separately in all other experiments, using 27.5% MHB as discrimination point (see also S3 Fig, S2 Data, and Materials and methods for determination of the cutoff point).

Under mild hyperpolarizing conditions, SwiChR-induced hyperpolarization results in a migration speed reduction independent of the position at the MHB (Fig 3D–3H). To test if such a mild hyperpolarization is a strong signal in THN migration, we activated SwiChR every 10 min for 48 h. We found that cluster formation at the ventral end of the MHB was delayed, but over time THNs migrated a short distance (S4 Fig and S6 Video). This suggests that hyperpolarization is a regulator of THN migration, though probably not the only one acting on THNs at the MHB.

In contrast to hyperpolarization, depolarization every 10 min via ChR2 did not affect THN speed (Fig 3G and 3H). When the depolarization interval was increased to 1 min and 6.57 μs per pixel or 1.64 ms per cell, THNs migrated faster than control cells, irrespective of their position at the MHB (Fig 3D–3H). Yet even in these highly depolarizing conditions, cells reduced their speed towards the ventral side of the MHB (Fig 3G and 3H). This hints at the presence of regulatory mechanisms other than membrane potential, which are able to modulate the depolarization-induced cell speed increase in the ventral portion of the MHB. Interestingly, none of the optogenetic manipulations induced THNs to deviate from their usual route, showing that activity controls migration progress rather than directionality.

### ACh is a THN migration promoting factor

As the optogenetic experiments demonstrated that cell-autonomous activity is directly involved in regulating THN motility, we set out to identify the neurotransmitter systems that drive the migration.

We developed an assay that allows us to quantitatively evaluate pharmacological compounds for their effects on migration. In *atoh1a*:*KalTA/4xUAS-GFP* embryos, all THNs in the developing cerebellum appear labelled by GFP expression [4]. This allows the tracking of the whole population under different conditions. Six to 12 fluorescent embryos from 27 hpf to 34 hpf were embedded laterally to improve tracking of THNs along the MHB. The embryos were kept in their normal medium supplemented with different compounds (S2 Table, see also S7 Video). The cerebellum was imaged in 5 μm steps every 10 min for a duration of 4 h. Each condition was analyzed at least in triplicate (see S3 Table for N and statistical parameters, see also S8 Video). Next, cells were tracked and tissue-shift corrected as in the optogenetic experiments.

**Fig 4:**
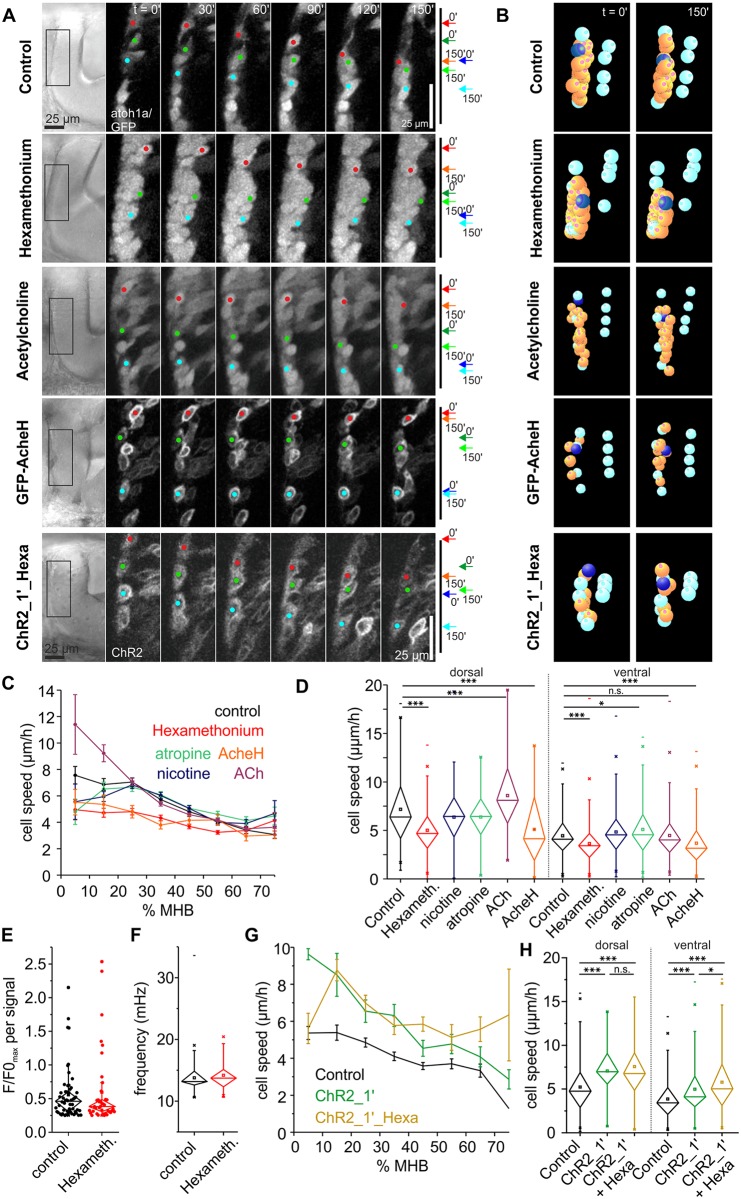
ACh promotes THN migration. (A) Tracking THNs in the *atoh1a*:*KalTA/4xUAS-GFP* fish line shows a migration decrease upon ACh receptor block, whereas stimulating THNs with excess ACh increases the migration speed in the dorsal portion of the MHB. Overexpressing AcheH mimics the effect of hexamethonium, whereas optogenetic depolarization rescues the hexamethonium speed decrease. THN progress over time is schematically indicated on the right. Elapsed time is indicated at the top. Scale bar: 25 μm. See also S7 Video. (B) Individual tracks (orange and dark blue) after shift correction (cyan) illustrate these effects. See also S8 Video. (C) Hexamethonium and AcheH overexpression slow cells down regardless of their starting point, whereas ACh has a stimulatory effect only in the dorsal region of the MHB. Neither atropine nor nicotine produce prominent effects. Means ± SEM are plotted per 10% MHB bin. (D) Dorsal (≤25% MHB) and ventral tracks (≥32.5% MHB) are similarly affected by hexamethonium or AcheH, whereas Atropine induces a slight speed increase only in ventral tracks. ACh increases speed in dorsally migrating THNs. Significance levels were calculated to control, and only significant differences are indicated. (E) Maximum values for each signal amplitude of F/F0 traces was plotted to illustrate that hexamethonium tends to dampen signal strength. Note that the difference to control is not statistically significant, due to the low *N* of signals. (F) The frequency spectrum contained in the FFT does not change upon hexamethonium treatment. (G) THN speed values along the MHB in ChR2-activating conditions are very similar regardless of hexamethonium treatment. Means ± SEM are plotted per 10% MHB bin. (H) Statistical analysis confirms that hexamethonium treatment does not change the ChR2 effect. Bars are means ± SEM. Data depicted in the graphs can be accessed in S1, S2 and S3 Data. ACh, acetylcholine; AcheH, Ache glycosylphosphatidylinositol-anchored isoform H; *atoh1a*; *atonal 1a*; ChR2, channelrhodopsin; FFT, fast Fourier transform; F/F0, fluorescence over background; GFP, green fluorescent protein; Hexameth, hexamethonium; MHB, midbrain-hindbrain boundary; n.s., not significant; THN, tegmental hindbrain nuclei neuron.

First, we tested the ACh system, as mature THNs are cholinergic [29]. THNs migrating under control (1% dimethyl sulfoxide [DMSO]) conditions made good progress along the MHB (Fig 4A and 4B; *N* = 600 tracks/19 embryos). Quantification revealed that migration speed again decreases as THNs approach the ventral end of the MHB, matching results from the optogenetic experiments (Fig 4C and 4D and S3 Fig, S3 Data). Blocking nicotinic ACh receptors with 500 μM hexamethonium strongly decreased migration speed, irrespective of the starting point (Fig 4A–4D; *N* = 694 tracks/23 embryos; see S3 Fig for the determination of cutoff points for dorsally- and ventrally-located cells). This reduction in THN speed is unlikely to be due to a gross mis-organization of the actin cytoskeleton, as hexamethonium does not appear to introduce obvious changes in THN morphology (S5 Fig). The total number of calcium transients is also reduced by approximately 30% in *Tg(elavl3*:*Hsa*.*H2B-GCaMP6s)* embryos (9.31 × 10^−3^ signals per min, or 39 signals in total observation time of 53.4 h collectively from 161 nuclei out of 15 embryos, compared to 13.37 × 10^−3^ signals per min in control cells or 56 signals in total observation time of 69.8 h from 195 nuclei out of 15 embryos, using a 0.25 F/F0 amplitude cutoff that excludes low-level oscillations; see also S1 Data). Yet the effect is most prominent on the strongest signals as determined by a 0.5 F/F0 amplitude cutoff, reflected in a reduction of approximately 64% (1.91 × 10^−3^ signals per min in hexamethonium-treated embryos versus 5.25 × 10^−3^ in control embryos). This suggests that hexamethonium dampens, rather than abolishes, calcium transients (Fig 4E and S2 Fig). The prominent transient frequencies contained in the dataset as determined by the maximal amplitude values in the FFT analysis do not differ significantly from controls (Fig 4F); neither does the proportion of nuclei exhibiting low-amplitude oscillations around 16.7 mHz (53 out of 161 nuclei total, or 32.9). Long-term imaging also suggested that the speed reduction is a strong effect, as THNs under continuous hexamethonium treatment lagged behind controls in their progress along the MHB and cluster formation (arrows in S4 Fig, see also S15 Video). In sum, hexamethonium appears to modulate calcium transient strength in THNs, which could be related to its speed reduction effect on migrating THNs.

In order to further characterize the effects of the ACh system on migrating THNs, we also blocked muscarinic ACh receptors by atropine. This treatment had only a slight effect on THNs in the ventral portion of the MHB. The attempt to activate nicotinic receptors by nicotine had no impact on the cells (Fig 4C and 4D). As this could be due to the low solubility of nicotine in water, we also tested the effect of 1 mg/ml ACh on THN migration, as ACh is readily soluble in water. This agonist induced a speed increase in dorsal THNs (Fig 4A–4D, *N* = 1223 tracks/28 embryos). The absence of a stimulating effect in the ventral region at the MHB could be due to the increased presence of acetylcholinesterase (AchE), which inactivates ACh [38,39]. Hence, we tested the effect of overexpressed AchE on THN motility. We hypothesized that the overexpression, or premature expression, of AchE in THNs could mimic the migration inhibition by hexamethonium via efficient removal of ACh from the immediate surroundings of the THNs. In order to restrict the effect of AchE to the THNs, we used a GFP-tagged version of an isoform of human AchE, known as Ache GPI-anchored isoform H (AcheH), which is anchored to the cell surface by glycosylphosphatidylinositol (GPI) modification ([40]; S3 and S5 Figs). Cell-type–specific overexpression was achieved by injecting the GFP-AcheH construct under 5xUAS control into *atoh1a*:*KalTA* embryos. As shown in Fig 4A–4D, overexpression of this construct induces a strong decrease in migration speed, similar to hexamethonium (*N* = 319 tracks/24 embryos). This demonstrates that ACh is indeed an important regulator of THN migration, acting directly on the THNs in a cell-autonomous manner.

Finally, we attempted to show that THNs express genes that play a role in the perception of ACh. As nicotinic ACh receptors are composed of five different subunits, each of which is represented by a large gene family in zebrafish, we decided to localize RIC3 acetylcholine receptor chaperone b (*ric3b*) and *ache* by whole mount in situ hybridization (WISH) instead. Ric3b is a chaperone involved in folding a number of various nicotinic ACh receptors [41,42]. *Ric3b* is widely expressed in the brain at 30 hpf (S3 Fig), especially in the ventral region of the hindbrain and the cerebellum. Staining is also visible at the MHB, and lighter staining in the cerebellar tissue. Similar results were obtained for AchE, with additional, very strong staining in the muscles as expected (S3 Fig).

Taken together, these results suggest that ACh-mediated activity is the source of regular depolarization for THNs. Therefore, we next asked whether ChR2-mediated depolarization can compensate for the loss of ACh-mediated depolarization. We accordingly depolarized THNs that were kept in 500 μM hexamethonium optogenetically at 1 min intervals as in the previous experiments (Fig 4A and 4B). Under these conditions, THNs migrate with increased speed, the depolarization substituting for the loss of ACh input (*N* = 535 tracks/17 embryos; Fig 4G and 4H, S2 Data). These results support the notion that depolarization is the primary migration-promoting signal, and it is normally achieved by ACh.

### Glycine negatively influences THN migration

**Fig 5:**
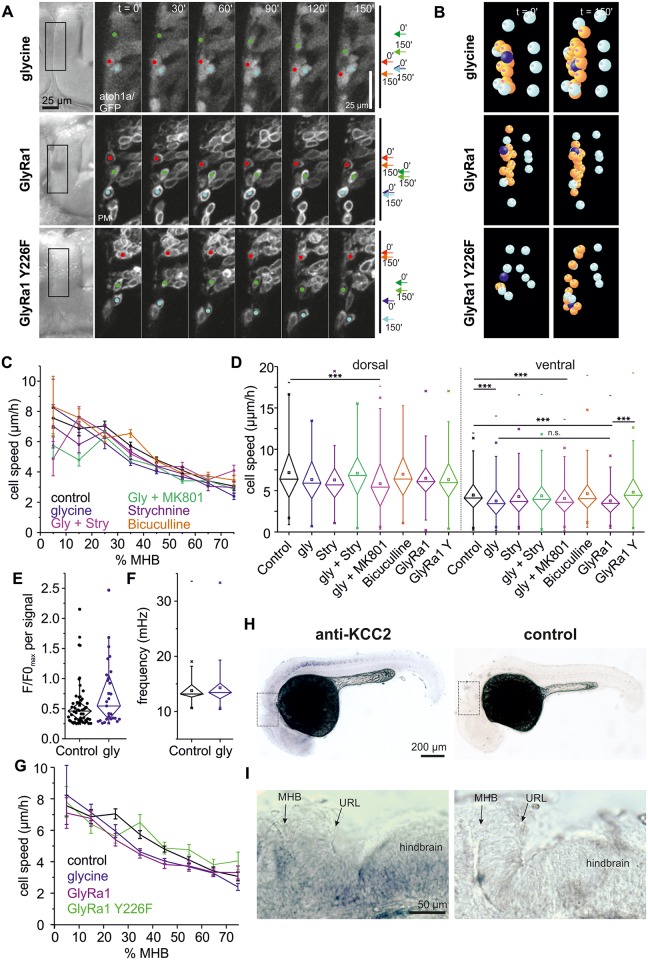
Glycine negatively influences THN migration. (A) Single cell tracking along the MHB shows that glycine as well as overexpression of a glycine receptor subunit slows THNs down. For control, a point mutation was introduced in the GlyRa1 subunit that abolishes ligand binding (Y226F). Their progress is schematically indicated on the right. Elapsed time is indicated at the top. Scale bar: 25 μm. See also S9 Video. (B) Glycine-treated THNs, as well as GlyRa1 wt or Y226F overexpressing THNs (orange, dark blue), were tracked in SIMI°BioCell software. References for correction are shown in cyan. See also S10 Video. (C) Glycine effects are strongest in the central region of the MHB. For control, embryos were treated with glycine together with MK801 or strychnine. Control values from experiment shown in Fig 4C. Means ± SEM are plotted per 10% MHB bin. (D) Statistic analysis confirms that the effects on THN migration speeds induced by glycine occur in the central region of the MHB and depend on the glycine receptor; control values from experiment shown in Fig 4D. (E) Calcium transient rates decrease in THNs upon exposure to glycine, but their strength does not differ from controls. Maximal F/F0 values from each transient are compared to controls from Fig 2G, and are statistically not significant. (F) FFTs contain frequencies of a similar spectrum compared to controls from Fig 2I. (G) Overexpression of the wt GlyRa1 subunit mimics the effects of excess glycine without adding extracellular glycine. When substrate binding is abolished (Y226F), THNs migrate similar to untreated control cells. Control values from experiment shown in Fig 4C and 4D. Means ± SEM are plotted per 10% MHB bin. (H) The hyperpolarizing effect of glycine depends on the co-expression of the channel KCC2. This gene is very weakly expressed in developing neurons as reported previously [45,46], but could be observed in the spinal cord and the hindbrain of a 30 hpf wt embryo (top panel, bottom panel: sense probe for control). Boxes indicate regions shown in (G). Scale bar: 200 μm. (I) Magnification of region indicated in (F) to show that KCC2 may be present at the MHB and the ventral region of the rostral hindbrain at 30 hpf, although its expression is weak. Scale bar: 50 μm. See also S5 Fig. Data depicted in the graphs can be accessed in S1 and S3 Data. F/F0, fluorescence over background; FFT, fast Fourier transform; GFP, green fluorescent protein; Gly, glycine; GlyRa1, glycine receptor alpha1 subunit; hpf, hours post fertilization; KCC2, solute carrier family 12 (potassium/chloride transporter), member 5b; MHB, midbrain-hindbrain boundary; MK801, dizocilpine; n.s., not significant; PM, plasma membrane; Stry, strychnine, THN, tegmental hindbrain nuclei neuron; URL, upper rhombic lip; wt, wild-type; Y226F, glycine receptor alpha1 subunit Y226 mutated to F.

The optogenetic experiments indicated that hyperpolarization negatively influences THN migration. This prompted us to screen for neurotransmitter systems that could modulate THN migration by hyperpolarization. We exposed embryos to either 200 μM Bicuculline to block GABA receptors, or 10 mM glycine to activate the glycine receptor. On THN migration, bicuculline exhibited no effect, but glycine slowed THNs down, although the effects appeared to be subtle (Fig 5A and 5B). After quantification, the effect of glycine on THNs is smaller than the one imposed by hexamethonium (Fig 5C and 5D, *N* = 748 tracks/20 embryos; see S3 Table for *N* and statistic parameters, S3 Data and S9 and S10 Videos), yet is similar to the speed reduction induced by optogenetics (Fig 3H). The effect is strongest in the central part of the MHB, between 20% and 50%, which is the critical region where most THNs initiate axonogenesis and nucleokinesis [4] (Fig 5C and 5D, and S3 Fig, S3 Data). Again, no morphological changes caused by glycine were detected (S5 Fig). In order to rule out that the effect of glycine is the result of its co-activating N-methyl-D-aspartate (NMDA) receptors, we repeated the analysis on embryos treated with 10 mM glycine, as well as either 50 μM dizocilpine (MK801) to block NMDA channels or 10 μM strychnine, which inhibits the glycine receptor (Fig 5C and 5D; S3 Data, *N* [MK801] = 682 tracks per 18 embryos, *N* [strychnine] = 357 tracks per 13 embryos). The speed reduction effects of glycine disappeared only upon strychnine treatment (*N* = 438 tracks per 16 embryos), proving that glycine acts via the glycine receptor.

In addition, if glycine acts directly on THNs via the glycine receptor, a change to the observed calcium transients in *Tg(elavl3*:*Hsa*.*H2B-GCaMP6s)* embryos should be observed. When using the most sensitive cutoff for signals (0.25), glycine reduces the rate of calcium transients by approximately 58% (5.49 × 10^−3^ signals per min or 27 signals from 256 nuclei per 15 embryos and a total observation time of 81.9 h, compared to control 13.12 × 10^−3^ signals per min or 56 signals from 195 nuclei per 15 embryos and a total observation time of 69.8 h; see also S2 Fig, S2 Data). Similarly, the maximal amplitude values for the remaining transients spread over a large range (Fig 5E), suggesting that glycine-induced hyperpolarization blocks transients from arising rather than modulating their strength. Regular calcium oscillations below the cutoff level appeared largely unaffected as seen in the FFT frequency analysis (Fig 4F) and by the proportion of nuclei with dominant frequencies in the 16.7 mHz range of 27.0%, or 69 nuclei out of 256 total number (control: 32.3%).

In all these experiments, embryos were exposed to high concentrations of glycine, and pharmacological treatments make it difficult to distinguish between direct and indirect effects of glycine on THNs. In order to prove that glycine is perceived by THNs, we overexpressed glycine receptor alpha1 subunit (GlyRa1) in the THNs. *Glra1* encodes for the alpha1 subunit found in many glycine receptors. As glycine receptors are composed of only alpha and beta subunits, we hypothesized that the overexpression of alpha subunits would only result in the formation of functional receptors when THNs endogenously express beta subunits. If functional glycine receptors are in this way overproduced in the THNs, it could increase their susceptibility to endogenous glycine (Fig 5A and 5B). For control, we expressed a point-mutated version (glycine receptor alpha1 subunit Y226 mutated to F [Y226F]) of GlyRa1, which is thought to abolish glycine binding [43,44]. Overexpressing this mutated protein should not result in increased susceptibility to endogenous glycine, and fully functional receptors in these THNs derive from the endogenously expressed proteins. After tracking THNs and calculating their speeds (*N* [GlyRa1 wild-type (wt)] = 347 tracks per 25 embryos, *N* [GlyRa1 Y226F] = 270 tracks per 18 embryos; see also S3 Data), we found that the overexpression of wt GlyRa1 is sufficient to slow THNs in the central part of the MHB in the absence of exogenous glycine to a similar extent as a high dose of glycine on normal THNs does (Fig 5D and 5G). The THNs overexpressing the point-mutated version behave like control cells.

The ability of glycine to hyperpolarize cells depends on the co-expression of the ion channel solute carrier family 12 (potassium/chloride transporter), member 5b (KCC2) and the glycine receptor. As had been reported in the literature, KCC2 is very difficult to detect in young embryos because its expression increases with neuronal maturation [45,46]. Nevertheless, we succeeded in localizing KCC2 in a distinct pattern along the spinal cord (Fig 5H and 5I). Weak staining was observed in the ventral part of the hindbrain, as well as at the MHB (Fig 5H and S3 Fig), making it likely that glycine acts as hyperpolarizer in THNs at least at the MHB.

Taken together, these results indicate that glycine could act as an opponent to ACh in controlling THN migration by balancing depolarization against hyperpolarization, especially in the transition region at the MHB.

### Glutamate has a spatially restricted role in regulating THN migration

The THN tracking assay identified glycine and ACh as neurotransmitters that influence THN motility. Their effects recapitulated the results from optogenetic hyper- and depolarization.

During screening for modulators of THN migration, we also tested the glutamate system. Glutamate has been shown to promote the migration of diverse neuron types in other animal models [14,15]. Glutamate acts on neurons via several receptors, of which two types are best studied: α-amino-3-hydroxy-5-methyl-4-isoxazolepropionic acid receptor (AMPA) receptors can be activated by glutamate and inhibited by 6-cyano-7-nitroquinoxaline-2,3-dione (CNQX), whereas NMDA receptors are activated by NMDA or glutamate together with a co-activator, and are inhibited by MK801.

**Fig 6:**
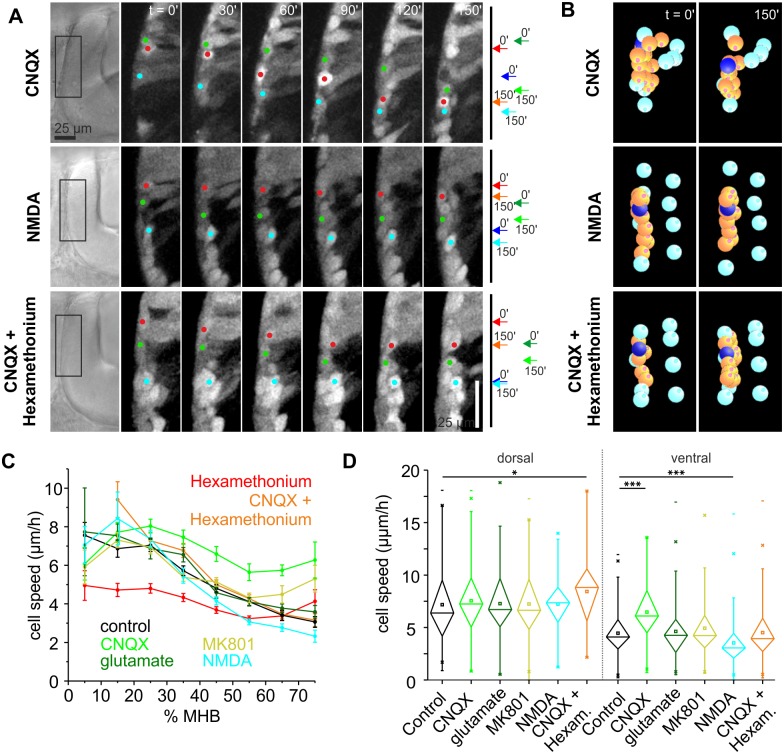
Glutamate negatively influences THN migration. (A) Following THNs along the MHB suggested a cell speed increase upon AMPA receptor block, whereas NMDA causes a speed decrease. CNQX and hexamethonium applied simultaneously produced no speed change in ventral tracks. Progress over time is indicated. Elapsed time is given at the top. Scale bar: 25 μm. See also S11 and S13 Videos. (B) Individual tracks (orange, dark blue) are represented after tracking and correction (cyan markers) in SIMI°BioCell. See also S12 and S14 Videos. (C) Migration speed analysis shows that blocking AMPA receptors increases speeds strongly in the ventral part of the MHB. NMDA produces the opposite effect again in the ventral region. Blocking AMPA and nicotinic ACh receptors simultaneously leads to an increase in THN speed in dorsal tracks, whereas in the ventral part, the effects outbalance each other. Control values from experiment shown in Figs 4D and 6C. Means ± SEM are plotted per 10% MHB bin. (D) Statistic analysis confirms that the effects on THN speeds by the glutamate system are confined mostly to the ventral part of the MHB; control values from experiment shown in Fig 4E. Bars are means ± SEM. Data depicted in the graphs can be accessed in S3 Data. ACh, acetylcholine; AMPA, α-amino-3-hydroxy-5-methyl-4-isoxazolepropionic acid receptor; CNQX, 6-cyano-7-nitroquinoxaline-2,3-dione; Hexam, hexamethonium; MHB, midbrain-hindbrain boundary; MK801, dizocilpine; NMDA, N-methyl-D-aspartate; THN, tegmental hindbrain nuclei neuron.

Surprisingly, treating embryos with 20 μM CNQX results in an increase in THN speed (Fig 6A and 6B). Moreover, the strongest effect occurred in the ventral part of the MHB where THNs normally slow down to settle (Fig 6C and 6D, see also S11 and S12 Videos, S3 Data). No morphological changes were detected upon CNQX treatment (S5 Fig). When THNs were exposed to CNQX over extended periods, cluster formation at the ventral end of the MHB appeared prematurely (S4 Fig). Next, we attempted to activate the glutamate system by adding 1 mM glutamate. However, we were unable to induce a slowdown in migration (Fig 6C and 6D). As we chose a comparatively low concentration of glutamate to avoid excitotoxicity [13], and glutamate is readily taken up by glial cells in zebrafish [47], it is possible that the concentration of glutamate in the tissue was too low to produce an effect. Therefore, we simulated the response to glutamate via activating NMDA receptors by adding 300 μM NMDA. This led to a THN migration speed decrease (Fig 6A–6D) in the ventral portion of the MHB (Fig 6C and 6D). Moreover, in several embryos that were treated with NMDA, we could observe highly protrusive, poorly polarized cells in the ventral region of the MHB (S5 Fig) where control cells are unipolar. For control, we also added 50 μM MK801 to block NMDA receptors, which had no effect on THN migration (Fig 6C and 6D). Taken together, glutamate acting via AMPA receptors is a slowdown signal to THNs near their terminal place of differentiation.

Although ACh and glutamate are both considered depolarizing neurotransmitters, they produce opposite effects on THNs. This led us to test whether glutamate and ACh could counteract each other. We added 20 μM CNQX together with 500 μM hexamethonium to the embryos and repeated the analysis. The drugs did counteract each other in the ventral region of the MHB to reach control levels (Fig 6A–6D, see also S13 and S14 Videos), suggesting that the glutamate and ACh responses are finely balanced. Surprisingly though, in the dorsal region—where only hexamethonium had shown a strong effect on THNs—THNs migrated faster, similar to THNs treated with CNQX alone (Fig 6F). This suggests that the glutamate-induced migration-blocking effect is more important to dorsal-situated cells, indicating that a slowing down in that region is crucial. This effect could be outbalanced by ACh over time, as THNs that continue to differentiate express more ACh receptor [29]. Testing this hypothesis, as well as answering the question how glutamate causes the speed reduction along the dorsoventral extent of the MHB, which was detected in all conditions, is an intriguing starting point for future investigation.

## Discussion

Previous studies using slice, implant, or in vitro cultures of neurons to investigate neurotransmitter-mediated activity in the context of migration had hinted that neurotransmitters can concertedly act on neurons to regulate migration [25,31,48]; e.g., a dualism between a hyperpolarizing and a depolarizing neurotransmitter had been described for hippocampal neurons and cortical interneurons [24,26,27]. Most of these assays, however, follow neurons in a partially or completely disrupted environment. This makes it difficult to interpret results in the context of in vivo migration in which additional stimuli affect neurons that are difficult to reconstitute artificially. Hence, we used live zebrafish embryos as models to study THN migration in vivo with minimal invasiveness.

We find that modulating the polarization state of THNs is a well-tuned regulator of in vivo migration as THNs pursue their normal route through the cerebellum. Using a combination of advanced quantitative track analysis and optogenetic tools, we demonstrate that depolarization increases THN motility, whereas hyperpolarization reduces THN migration speed. We developed a screening method for compounds that act in neuronal migration, and identified ACh as the likely source of depolarization of THNs, whereas glycine hyperpolarizes THNs to reduce migration. Glutamate has additional, spatially defined negative effects on migration that counterbalance the depolarization by ACh.

**Fig 7:**
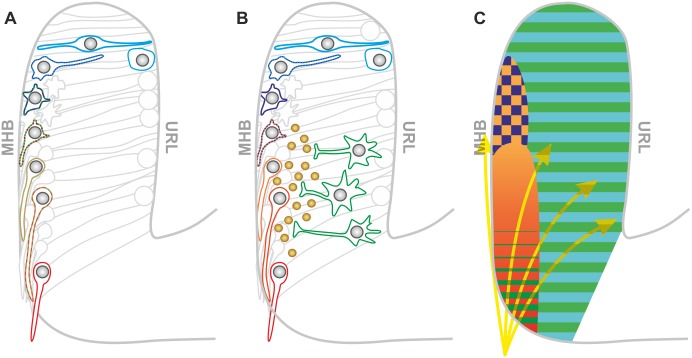
Model: Activity generates a map to regulate THN migration. (A) THNs respond to different neurotransmitter systems successively along their route. First, THNs react to glycine (blue). At the MHB, cells need to orient ventrally. In order to avoid that THNs move in the wrong direction at this stage, a slowdown is imposed by glycine-induced hyperpolarization and glutamate. As THNs have established the new direction and begin axonogenesis [4], they respond to ACh (orange to red). ACh-mediated depolarization counteracts the hyperpolarization by glycine to promote migration in phase 2. Glutamate is now perceived by the THNs to slow down in the ventral part of the MHB as they approach their destination. (B) Alternatively, THN migration could be regulated by glycine-induced hyperpolarization and ACh-mediated depolarization as outlined in (A). Yet, as glutamate also promotes depolarization, it is possible that glutamate acts on a second, as yet unidentified group of neurons (green cells) that releases THN-inhibiting factors (brown spheres). (C) Map of the cerebellum of a 28 hpf embryo, charting the regions of influence for different neurotransmitter systems. In this map, glutamate (green) and glycine (blue) are present throughout the cerebellum, whereas ACh is present at the MHB. Their perception is likely to depend on the THNs expressing the respective receptors. The overlap of glycine and ACh occurs in a region spanning approximately 25%–35% MHB. The territories of influence for the neurotransmitters do not fully dictate the response by the THNs, as these also integrate additional influences, such as a chemical gradient from the ventral region of the MHB (yellow arrows), that function as guidance cues. ACh, acetylcholine; hpf, hours post fertilization; MHB, midbrain-hindbrain boundary; THN, tegmental hindbrain nuclei neuron, URL, upper rhombic lip.

The results from our study can be summarized in a comprehensive model incorporating all effects (Fig 7A); several neurotransmitter systems act on THNs as they migrate from the URL towards the ventral end of the MHB, and postmitotic THNs emerging from the URL are influenced by glycine and glutamate [49–51]. As THNs reach the MHB, they undergo a critical change in migration type, morphology, and differentiation. Distel et al.[4] demonstrated that axonogenesis begins when THNs reach the MHB, and correct axon orientation is crucial for future functionality of the neuron. Typically, patterns of chemical attractants and repellants guide the axon [10]. Although chemical cues guiding THNs have yet to be identified, the direction of axon outgrowth never changed upon interference with activity in our study, making it likely that the determination of migration direction relies on other cues. A temporary slowing down of THNs to facilitate transition and pathfinding could be introduced by glycine-mediated hyperpolarization at the MHB. This is counteracted by increased depolarization by ACh when THNs move further ventral. As mature THNs are cholinergic [29], the migration-promoting effect of ACh would lead to a strong migration speed increase near their destination where THNs need to stop, due to the increased expression of ACh receptors. Therefore, glutamate is used as a restraint. Glutamate could either be perceived by the THNs directly or by a second, as yet unidentified, neuronal population that releases an inhibitory factor (Fig 7B). As optogenetic depolarization leads to a strong THN speed increase, and glutamate-mediated depolarization produces the opposite effects on the cells, we think the latter model to be more likely. A similar interaction of two neuron populations during migration has recently been described in mice [52]. Still, as the perception of glutamate is complex and may go beyond membrane depolarization, e.g., via metabotropic glutamate receptors, we are currently not able to distinguish between the two models.

The concerted action of the neurotransmitters can be summarized as a map of the developing cerebellum (Fig 7C) in which the neurotransmitters mark out territories of influence. Overall, none of the neurotransmitters was able to impose a complete stop, or cause over-migration of THNs, arguing that neurotransmitters are an important modulator of migration, but not the only one. THNs are likely to integrate input also from nonactivity-related sources, such as chemical cues, extracellular matrix changes, or mechanosensory influences at the MHB. They also respond to internal parameters such as maturation stage, so that the overall effect of the polarization state on migration is determined by the individual THN.

In the course of this study, we were surprised to find that even mild hyperpolarization has a clear effect on THN migration. This was demonstrated in optogenetic experiments as well as in the overexpression of only one subunit of a glycine receptor. This suggests that the cells are highly sensitive to changes in their polarization state, and that low-level activity could be sufficient to regulate migration. In this respect, the low levels of calcium transients that we detected in the THNs could be very meaningful in the context of determining migratory behavior. Technological improvements will help with the further analysis of calcium transients in the future. Of particular interest will be a correlation of the different calcium transient patterns that we identified with the migratory stages of individual THNs over extended observation periods, following the neurons in their 3D migration through the tissue. Likewise, the role of the regular calcium oscillations that we found in a subset of neurons remains unsolved as present, but becomes even more interesting in light of their independence from the ACh- and glycine-mediated effects.

Still, neurotransmitters could make an important contribution to ensuring that the neurons arrive at their destination in an orderly fashion in time, even without having the ability to fully block migration. As THNs migrate during a defined developmental window between 24 hpf and 48 hpf [29], cells that have failed to reach their destination during this time are likely to become trapped permanently in the wrong environment. They could come into contact with granule cells of the cerebellum, the next neuronal population to emerge from the URL that follows a very similar route [30]. This could result in the formation of interactions between the cell types that would not occur in the normal situation. The consequences of this could be dramatic for the whole organism, and will be an important research area in the future.

Our study demonstrates that different neurotransmitters antagonize each other in regulating migration in vivo. A similar counteracting balance between glutamate and GABA has been reported for cortical neurons [24]. Our regional tracking analysis revealed that ACh and glutamate, and most likely also ACh and glycine, have distinct regions at the MHB where they interact to produce a fine-tuned regulation of migration progress. The successive action of different neurotransmitters in cortical neurons controls their ability to traverse various layers [24]. As THNs do not cross distinct layers along their route, the successive action of neurotransmitters appears to be related to aligning maturation stage to migratory progress, e.g., by maintaining a temporary halt to migration until axonogenesis has been established. In this respect, it seems that successive and antagonistic neurotransmitter-mediated activity is a fundamental, but versatile, principle to control migration that is conserved across vertebrates.

Our THN in vivo migration model is going to provide us with more fundamental, transferable insights into neuronal cell migration on the cellular and subcellular level in the future. Ultimately, it will add to our understanding about how neuronal layers and nuclei are properly positioned in a spatiotemporal pattern.

## Materials and methods

### Ethics statement

All procedures involving animals were carried out according to EU guidelines and German legislation (EU Directive 2010_63, licence number AZ 325.1.53/56.1-TU-BS).

### Experimental model

Zebrafish were housed in groups of 20–40 individuals in a fish facility (Aquaneering, San Diego, CA) maintaining around 700 mS, pH 6.9–7.1 and 28°C under a 14/10 h light/dark cycle in accordance with common zebrafish handling guidelines [53]. All experiments involving live cell imaging were carried out using fertilized eggs from the fish line Tg(*atoh1a*:*Gal4TA4)* (line hzm2Tg) [4], crosses of Tg(*atoh1a*:*Gal4TA4)* with Tg(*4xUAS-GFP)* (hzm3Tg) [54], or Tg(*elavl3*:*Hsa*.*H2B-GCaMP6s)*,jf5Tg; [32]. Wt brass embryos were used for WISH. All embryos were kept in egg water (0.03 g/l sea salt) for 1 h before incubating the eggs in 30% Danieau medium (0.12 mM MgSO_4_, 0.21 mM KCl, 0.18 mM Ca(NO_3_)_2_, 17.4 mM NaCl, 1.5 mM HEPES, pH 7.2). To avoid pigmentation, 150 μM PTU was added to the 30% Danieau medium at 8–12 hpf. All embryos were incubated at 28°C. For imaging, normally developed embryos of similar fluorescence were selected from the same clutch.

### Method details

#### Plasmid construction

For calcium imaging, a bidirectional vector was created to express GCamp6s-CAAX and FyntagRFP-T under control of a 5xUAS element and E1b promoters (#3656). The vector included flanking Tol1 sites for genomic integration. A vector containing these repeats, as well as E1b 5xUAS E1b FyntagRFP-T, was opened with EcoRI and NotI to insert GCamp6s-CAAX. GCamp6s-CAAX was generated by PCR product over the addgene plasmid #40753, with primers adding the C-terminus of zebrafish K-Ras [55], similar to reported methods [34].

Using this PCR product, a Tol1 5UAS E1b vector was created to express only GCamp6s-CAAX (#3655). This plasmid was opened by EcoRI and BsrGI to exchange GCamp6s for YFP. The resulting frameshift was removed by Klenow treatment at the BsrGI site. pTol1 5xUAS E1b YFP-CAAX (#4014) was used as control in optogenetic experiments.

The optogenetic constructs pTol1 5xUAS E1b SwiChR-YFP (#3878) and pTol1 5xUAS E1b ChR2-YFP (#3957) were created by opening a pTol1 5xUAS E1b GFP vector with EcoRI and BsrGI to exchange GFP for a PCR product over either addgene plasmid #55630 or #20945, respectively.

These plasmids were each opened with EcoRI and XhoI to release the 5xUAS E1b element to be replaced by the CMV promotor from the pCS vector, obtained by EcoRI/SalI digestion. These constructs (#4931 and #4932) allowed the Tol1-mediated genomic integration and universal expression of the optogenetic channels in Tg(*elavl3*:*Hsa*.*H2B-GCaMP6s)* embryos.

To complement these experiments in *atoh1a*:*KalTA*-posititive embryos, GCaMP6s was cloned in a pCS vector containing a nuclear localization sequence by EcoRI and XbaI (#3885). This fragment was released by HindIII and KpnI and ligated into pBluescript containing H2B-mRFP (#3956).

For overexpression of AcheH in *atoh1*:*KalTA*-positive embryos, human *acheH* was amplified by PCR from Biocat clone 105060 without the signal peptide, which was instead fused to GFP. These PCR products were subsequently combined, and the resulting SP-GFP-AcheH (in the manuscript referred to as GFP-AcheH) inserted into the EcoRI site of a vector containing Tol1 repeats, FyntagRFP, and E1b 5xUAS E1b (#4857).

A construct to overexpress wt GlyRa1 was prepared in the same way, except that the PCR was based on Biocat clone 162943 containing zebrafish *glra1* (#4836).

This plasmid was subsequently point-mutated to introduce the Y226F mutation (#4854), which is defective in binding glycine, based on results from [43,44].

For WISH probe construction, the 3ʹ half of *ric3b* (#4938), the 3ʹ end of *ache* (#4955), and *kcc2* (*slc12a5b*, #4366) were each amplified from zebrafish cDNA of 48 hpf wt embryos. *Ric3b* and *kcc2* were inserted into pBluescript via EcoRI and HindIII, *ache* by XhoI and XbaI.

#### Injection

For experiments that relied on tissue-specific transgene expression, fertilized eggs from *atoh1a*:*KalTA*^+/−^ or *elavl3*:*H2B-GCaMP6s* parents were injected with either 25 ng/μl plasmid DNA alone or 100 ng/μl plasmid DNA together with 100 ng/μl mRNA encoding for transposase Tol1, prepared by in vitro transcription, using an Eppendorf Femtojet Express Microinjector. All injection mixes contained 0.05% Phenolred (Sigma Aldrich, St. Louis, MO) for injection control. Plasmid DNA was prepared using the manufacturer’s instructions (Macherey Nagel GmbH & Co., Düren, Germany). Approximately 2 nl were injected per zygote. After injection, eggs were transferred to 30% Danieau and incubated at 28°C. Embryos used in optogenetic experiments were raised in the dark.

#### In vivo imaging

As the *atoh1a* promoter only becomes active from approximately 17 hpf, embryos were selected for imaging from 24 hpf using a stereofluorescence microscope (Leica M205FA or MDG41). For confocal imaging, the chorion was removed using forceps. All embryos were sedated for embedding by incubating them in 30% Danieau + 150 μM PTU + 0.02% Tricaine for 10 min at RT. Next, embryos were embedded in lateral position in 1.2% ultra-low gelling agarose (type IX-A, Sigma-Aldrich) in 30% Danieau. The embryos were imaged on a confocal microscope (Leica TCS SP8). Embryos observed for >6 h were imaged using a 20x immersion objective, shorter experiments on a 40x water objective. All experiments were carried out at 28°C using a Life Imaging Services heating chamber. Images were recorded at single z-planes in the case of calcium experiments, as z-planes of different step sizes in all other experiments to cover the cerebellum. Typically, these ranged from 2.5 μm to record cellular morphology to 5 μm in migration assays. Image acquisition was controlled by the LasX software (Leica Biosystems, Wetzlar‎, Germany).

#### Calcium imaging and analysis

In order to investigate calcium signals in the cerebellum during THN migration, embryos of >28 hpf either co-expressing GCamp6s and Fyntag-RFPT or H2B-GCaMP6s alone or together with ChR2/SwiChR were imaged by cLSM. Embryos were recorded continuously in a single z-plane for 30 min with a 436-ms frame interval. Total fluorescence intensity was measured in the region that a single nucleus traversed using the Time Series Analyzer plugin for ImageJ. In case the measured nucleus left this region, or another nucleus entered, only the duration that the nucleus occupied the region alone and in focus was included in the analysis. A baseline to correct the fluorescent traces for background fluorescence was calculated as outlined in [33]. The baseline value for each timepoint was calculated from a window of 100 values (10% lowest values as baseline). F/F0 traces were obtained by dividing the fluorescence value by the baseline. In order to remove noise, the traces were bandpass filtered (cutoff values 20 and 200 mHz). The signal was mapped to the frequency domain using FFT in order to be able to evaluate the frequency of signal components for their prominence. The MatLab-based scripts for this analysis are available from: https://github.com/tobiasring/zebrafish-fluorescence

For calcium map construction, images with marked positions were aligned by the tissue borders identified in transmitted-light images and projected into a single image. Ratiometric images illustrating the increase in intracellular calcium were generated using the Ratio Plus plugin for ImageJ, comparing the GCamp6s fluorescence in the subsequent (after calcium transient) or preceding (before calcium transient) frame after background subtraction. In false-colored images, white represents high difference and blue similar intensity values.

#### Chemicals

Neurotransmitter channel inhibitors and activators were prepared as stock solutions and kept at −20°C in single-use aliquots (S2 Table). Drugs were defrosted and dissolved in 30% Danieau medium right before use. Due to this application method, working concentrations in the medium were chosen at high levels, with the exception of glutamate, to avoid excitotoxicity.

#### WISH

WISH was performed based on the protocols by [56]. Briefly, wt embryos were fixed at 30 hpf in 4% paraformaldehyde/PBS ON at 4°C and stored in 100% methanol at −20°C until use.

Probes used in WISH were created by in vitro transcription from the T7 (sense) and T3 promoters (antisense) with DIG labelling mix (Roche) according to the manufacturers’ instructions. DNA was digested using DNase (Promega Corporation, Durham, NC).

After removal of methanol, embryos were permeabilized with 2% H_2_O_2_ and 10 μg/ml proteinase K. Permeabilization was stopped by incubation in 4% PFA in PBST for 45 min. Embryos were prehybridized by incubation in hybridization buffer (50% formamide, 4xSSC, 50 μg/ml heparin, 50 μg/ml torula RNA, 0.1% Tween-20) for 6 hat 65°C before adding the probe. Hybridization was allowed to proceed ON. Next, embryos were washed several times in 50% formamide in 2xSSC, 2xSSC, and 0.2xSSC at 65°C. Embryos were then preadsorbed by adding 10% normal goat serum in PBST. After 1-h incubation at RT, the HRP-coupled anti-DIG antibody (Roche) was added at 1:2,000 and embryos incubated ON at 4°C. For detection of the antibody, the embryos were washed several times in PBST before washing them in staining buffer (100 mM NaCl, 100 mM Tris pH 9.5, 50 mM MgCl_2_, 1% Tween-20). The solution was then changed to staining buffer containing 5 μg/μl NBT and 3.75 μg/μl BCIP. The reaction was stopped by washing the embryos several times in PBST. Embryos were then transferred to 90% glycerol and imaged using a Zeiss Leitz DM RBE fluorescence microscope equipped with a Nikon DS-Vi1 camera.

### Quantification and statistical analysis

Data arising from the quantification procedures described below and incorporated into the graphs depicted in the figures and supplemental figures can be found in S1–S3 Data.

#### Calcium transients

F/F0 traces were obtained and Fourier transformed as described above.

F/F0 traces were screened for calcium signals after noise reduction by application of a 20–200 mHz bandpass filter, using a 0.25 or 0.5 F/F0 amplitude cutoff. As 0.25 is a very sensitive cutoff level likely to produce false positives, these signals were confirmed by inspection of the original films. In order to determine frequencies contained in the traces, the FFTs were scanned for prominent peaks. A prominent peak was assigned when its amplitude was at least 1.25 times higher than the next highest value. For whole datasets, the frequency corresponding to the maximal amplitude value was determined for each nucleus and plotted in Figs 2, 4 and 5. In addition, all FFTs were screened for the presence of a frequency in the range of 15.0–18.4 mHz. Finally, if the signal component with the highest amplitude value of a complete trace of a nucleus fell within this range, it was considered a prominent signal.

As this outlined procedure included nuclei with very different signalling patterns, we specifically determined the oscillation frequency of the subset of nuclei that showed strong, regular oscillations in the following way: out of the total number of 195 control nuclei, 32 were selected by inspection of their F/F0 traces for the appearance of regular oscillations irrespective of amplitude strength. To the first prominent peak appearing in the FFT traces of these nuclei, a Gaussian curve was fitted. The center of this fitted curve determined the frequency value of the oscillation, reported in Fig 2I.

#### Optogenetic analysis

For optogenetic manipulation, embryos expressing either ChR2-YFP or SwiChR-YFP were generated as described. SwiChR is a mutated version of ChR2, which functions as Cl^-^ channel [36]. From 28 hpf, channels were opened using 15% of the 476-nm laser line to open the channels and 0.5%–1.5% of 514 nm to visualize the THNs. This corresponds to an illumination time of 14.77 μs per pixel or 3.15 ms per average-sized THN for all 10-min regular channel opening regimes, 6.57 μs per pixel, or 1.64 ms per cell for 1 min regimes. These settings were first applied to H2B-GCaMP6s or NLS-GCaMP6s/H2B-mRFP embryos co-expressing the respective optogenetic channel, and the GCaMP6s response to channel opening using these settings was recorded. These calcium traces, along with negative controls in the form of THNs expressing only one of the proteins, were processed as outlined above. Nuclei were scored as positively responding to the ChR2 stimulus when the Fourier transformation showed a clear peak at the expected frequency.

For migration control, embryos expressing PM-targeted YFP were imaged under the same illumination conditions. Cells were recorded in one-half of the cerebellum for 4 h, either at 10-min (YFP-CAAX, SwiChR, ChR2_10ʹ) or 1-min (ChR2_1ʹ) intervals.

The assay was repeated 3 to 4 times for each condition, and all cells expressing the constructs analyzed (for *N* of embryos and tracks, refer to S1 Table). Next, the cells were manually tracked using SIMI°BioCell software (SIMI reality motion systems, Unterschleißheim, Germany), which allows forward and reverse tracking. Only cells that could be reliably identified for a minimum of 30 min were tracked.

During imaging, the embryos continue their normal development, leading to a displacement of the cerebellar tissue. This shift is highly individual and needs to be corrected in order to compare tracks from different embryos. To this end, quantitative track analyses were carried out with “Phainothea” (https://github.com/IaniiNN/CMC). Eight markers were placed equally on the URL and the MHB. From these markers, a center of mass was calculated whose movement was subtracted from each cell track to correct for the shift of the cerebellum. Track distances were calculated using 30-min intermediate distances and bee lines calculated from the endpoints of each track. Details on the software and its use in tracking cells in live embryos can be found in [37]. In order to determine each track’s starting point, additional reference markers were placed at the dorsal and ventral end of the MHB (0% MHB and 100% MHB, respectively). These markers were subjected to the same correction method. The tracks from all embryos were then pooled in 10% bins by their starting point along the MHB, and means with SEM plotted in Figs 3–6. Sample size was considered adequate once significant differences to control values were found (see statistical analysis section). For conditions with similar *N* values without significant differences to controls, an increase in sample size was considered unproductive, and changes to the assay conditions were introduced.

#### Screening assay and track analysis

In order to screen inhibitors and activators for different neurotransmitter systems, embryos of the fish line *atoh1a*:*KalTA/4xUAS-GFP* were selected for similar expression levels at around 24 hpf. At 27 hpf to 34 hpf, embryos were embedded for cLSM analysis. The embryos were kept in 30% Danieau medium supplemented with 150 μM PTU and 0.01% Tricaine, with the relevant concentration of the drug under investigation. Embryos were immediately transferred to the microscope. The cerebellum was imaged in 5 μm steps every 10 min for 4 h. Tracks were analysed as described in the optogenetic analysis section.

The assay was repeated 3 times for each condition investigated (for *N* of embryos and tracks, refer to S3 Table). The tracks from all cells were pooled, means with SEM plotted in Figs 3–6 and conditions tested for significant differences to control values. Test conditions were considered adequate for compounds that showed significant differences to control. For conditions with high *N* values without significant differences, an increase in sample size was considered unproductive under the given assay conditions.

#### Statistical analysis

Optogenetic and compound screening analysis experiments were carried out at least in triplicates. Tracks from all analysed embryos were classed as dorsal or ventral by their starting point. To identify a transition point, data were tested for the quality of their linearity using the Pearson’s coefficient in a series of 2.5% MHB decreases or increases. This analysis showed that there is a transition area between 25% and 32.5% MHB in control THNs treated with 1% DMSO in which linearity is poor (Pearson’s coefficient drops). For YFP-CAAX control cells used in optogenetic experiments, no clear transition region emerged using this method, but instead suggested 27.5% as transition point. Hence, a starting point <25% MHB signifies dorsal, >32.5% ventral in drug screening experiments; for optogenetic experiments, a 27.5% discrimination point for dorsally- and ventrally-located THNs was employed.

All tracking data distributions were analysed for their Gaussian distribution using a Shapiro-Wilk test, and significance levels calculated using a nonparametric Kruskal-Wallis-ANOVA test using Origin software. All conditions were tested against control levels, which is YFP-CAAX for optogenetic experiments and 1% DMSO treatment for the compound screening analysis. In addition, speeds from THNs overexpressing GlyRa1 wt were compared to the mutated GlyRa1 Y226F expressing cells. Significance levels that were tested are *p* < 0.05, *p* < 0.01, and *p* < 0.001. S1 Table lists *p*- and chi-squared values for optogenetic experiments described in Figs 3H and 4H; S3 Table shows values for compounds used in the screening assay described in Figs 4D, 5D and 6D.

#### Software for image processing

Images were processed for brightness and contrast, cropped and annotated using ImageJ/FIJI, SIMI°BioCell, and Adobe Photoshop software packages. In particular, for image presentation in all figures and supplemental videos, individual frames from films were realigned using the rigid body method included in the MultiStackReg plugin for ImageJ. Calcium fluorescence was measured using the Time Series Analyzer plugin for ImageJ, and analysed using Matlab scripts developed by [33] for baseline correction, and by TR for denoising and frequency analysis. Tracks were measured in SIMI°BioCell (SIMI reality motion systems) and analysed with “Phainothea” (https://github.com/IaniiNN/CMC). Data were statistically analyzed and plotted using Origin and Microsoft Excel software. Figures were arranged in CorelDraw.

## Supporting information

S1 Fig (A) Left: Full kymograph of the GCaMP6s-CAAX recording from the example THN shown in Fig 2J. Box indicates the region magnified on the right. Calcium transients are indicated by arrows. Vertical scale bar: 20 s, horizontal: 10 μm. (B) Signaling THNs were assigned to three regions in the cerebellum, but no regional preference for calcium transients was detected. (C) Low calcium frequencies detected with the PM-bound calcium sensor match results obtained from *elavl3*:*H2B-GCaMP6s* embryos. (D) Calcium transients show great variability in their duration. (E) Maximum signal intensity of GCamp6s from the example THN in (A) plotted by frame. Fyn-tagRFP-T fluorescence intensity remains similar over time, fluorescence increases in GCamp6s therefore are not due to position shifts. Intensities were measured along a line indicated in image. Intensity values were averaged over 5 neighboring points. (F) In about one third of all THNs, calcium transients occur at the front. CAAX, PM-targeting signal derived from K-Ras; GCaMP6, circular permutated green florescent protein-Calmodulin-M13 peptide 6; PM, plasma membrane; THN, tegmental hindbrain nuclei neuron.
(TIF)

S2 Fig (A) Overview of the cerebellum of a Tg*(elavl3*:*Hsa*.*H2B-GCaMP6s)* embryo expressing SwiChR-YFP. Box indicates the nuclei whose calcium traces are given in (B). Anatomical features are indicated. Scale bar: 25 μm. (B) Example calcium F/F0 traces of SwiChR-expressing Tg*(elavl3*:*Hsa*.*H2B-GCaMP6s)* embryos. Note that the bottom trace was obtained from a cell that expressed only H2B-GCaMP6s and serves as negative control. Fourier transformed traces of the same examples are given on the right. None of the traces show a peak except for frequencies in the range of the endogenous oscillations. The blue light illumination of SwiChR would correspond to a 1.67 mHz frequency (5 nuclei expressed both markers in 2 embryos). (C) Overview of the cerebellum of a Tg*(atoh1a*:*KalTA)* embryo co-expressing NLS-GCaMP6s/H2B-tagRFP and ChR2-YFP. Box indicates the nucleus whose calcium traces are given in (D) and (E) as nucleus 1. Anatomical features are indicated. Scale bar: 25 μm. (D) Examples of calcium F/F0 traces of ChR2 or SwiChR-positive THNs co-expressing NLS-GCaMP6s. Only the nuclei with ChR2 show strong regular peaks (arrows; 9 nuclei out of 36 measured including controls from 9 embryos). In SwiChR, occasionally some nuclei respond to endogenous signals (top). These events are too rare to be noted in the Fourier transformation (see (E); 13 nuclei from 13 embryos expressed both markers). Note that the bottom trace in SwiChR is derived from a cell that expressed only NLS-GCaMP6s, but no SwiChR, and serves as negative control. (E) Fourier transformed traces of the examples given in (D). Nucleus 1 of the ChR2 traces shows a clear peak at the expected frequency of 16.7 mHz. SwiChR-derived traces do not show such a peak except for frequencies in the range of the endogenous oscillations. The blue light illumination of SwiChR would correspond to a 1.67 mHz frequency. (F) Example traces from Tg*(elavl3*:*Hsa*.*H2B-GCaMP6s)* embryos treated with hexamethonium demonstrate that calcium signal amplitudes are decreased (left). FFTs of these traces are given on the right. (G) Example traces from Tg*(elavl3*:*Hsa*.*H2B-GCaMP6s)* embryos treated with glycine demonstrate that calcium signal amplitudes are not affected by the hyperpolarizing agent (left). Note that these examples are chosen from the minority of nuclei that still exhibited calcium transients. FFTs of these traces are given on the right. ChR2, channelrhodopsin; F/F0, fluorescence over background; FFT, fast Fourier transform; GCaMP6, circular permutated green florescent protein-Calmodulin-M13 peptide 6; H2B, Histone 2B; NLS, nuclear localization sequence; SwiChR, mutated channelrhodopsin; YFP, yellow fluorescent protein.
(TIF)

S3 Fig (A) Speeds from control THNs expressing YFP-CAAX migrating under the same illumination conditions as used in the optogenetic experiments are given as individual dots. Red lines represent the regression lines based on all values below or over the cutoff point 27.5% MHB. (B) Speeds from control THNs expressing GFP treated with 1% DMSO are given as individual dots. Red lines represent the regression lines based on all values below 25% MHB or over the 32.5% MHB. (C) THNs migrating under control conditions (1% DMSO) slow down from 25%–35% MHB as they progress ventrally. Track distribution is indicated at the top. (D) At high resolution, GFP-AcheH (top panel) appears to be present inside THNs as well as at the PM (middle). The overall morphology of these early phase 2 THNs appears unaffected. Scale bar: 10 μm. (E) WISH of a 30 hpf wt embryo stained for *ric3b* expression. This chaperone for nicotinic ACh receptors shows a strong expression in the brain. Box indicates the region magnified in the lower panel. Prominent anatomical features are indicated. Scale bars: 200 μm/50 μm. (F) Sense probe against *ric3b* is used as control for the staining shown in Figure S3E. Box indicates the region magnified in the lower panel. Major anatomical features are indicated. Scale bars: 200 μm, 50 μm. (G) WISH against *ache* shows strong staining in the muscles, the ventral hindbrain and a weaker staining in the cerebellum. This is more clearly seen in the magnified region in the second image. Anatomical features are annotated. Scale bars: 200 μm/50 μm. (H) As control, the sense probe against *ache* was used. Major anatomical features are indicated. Boxes indicate the magnified regions. Scale bars: 200 μm/50 μm. (I) Plots of every track in the glycine dataset along the MHB do not indicate the presence of subpopulations of THN cells with differential responses to glycine. The overall distribution of the tracks is indicated at the top. (J) *Kcc2* (*slc12a5b*) is weakly expressed in the cerebellum at 30 hpf. Stronger staining can be observed in the ventral hindbrain and the spinal cord; image on the right is magnified region indicated in the left image. Prominent anatomical features are indicated. Scale bars: 100 μm/ 50 μm. (K) Sense probe was used as control for WISH against *kcc2* (*slc12a5b*); image on the right is magnified region indicated in the left image. Prominent anatomical features are indicated. Scale bars: 100 μm/ 50 μm. Data depicted in the graphs can be accessed on the PLOS Biology Supplemental Information webpage (S2 and S3 Data). ACh, acetylcholine; AcheH, Ache GPI-anchored isoform H; CAAX, PM-targeting signal derived from K-Ras; DMSO, dimethyl sulfoxide; GFP, green fluorescent protein; hpf, hours post fertilization; MHB, midbrain-hindbrain boundary; PM, plasma membrane; THN, tegmental hindbrain nuclei neuron; WISH, whole mount in situ hybridization; YFP, yellow fluorescent protein.
(TIF)

S4 Fig (A) Fluorescent images are overlaid on transmitted light images to show tissue development and delayed THN migration progress in prolonged hyperpolarization. Arrowheads indicate the starting points, arrows point to the THNs’ progress. Stars indicate the original starting point after THNs have left the region. Developmental stages are indicated. Scale bar: 100 μm. See also S6 Video. (B) Magnified cerebellar region from images of control THNs shown in Fig 1B. THNs emerge from the URL, then follow the MHB to form a cluster at the ventral end of the MHB, indicated by magenta line. Yellow arrow indicates the dorsal end of the cerebellum to facilitate the localization of THNs in the tissue. Developmental stages are indicated at the top. Scale bar: 20 μm. (C) Prolonged hexamethonium treatment leads to a delay in THN cluster formation as seen by the elongated distribution of THNs instead of a cluster (magenta outline). The arrow indicates the dorsal end of the cerebellum and THNs that are still distributed along the MHB. See also S15 Video. (D) Blocking AMPA receptors continuously with CNQX leads to premature aggregation of THNs near the ventral region of the cerebellum. Note the size and shape of the cluster at 60 hpf (outlined in magenta) compared to control in (B). Arrow denotes the dorsal end of the tissue. AMPA, α-amino-3-hydroxy-5-methyl-4-isoxazolepropionic acid receptor; CNQX, 6-cyano-7-nitroquinoxaline-2,3-dione; MHB, midbrain-hindbrain boundary; THN, tegmental hindbrain nuclei neuron; URL, upper rhombic lip.
(TIF)

S5 Fig (A) Control THNs treated with 1% DMSO exhibit the typical morphology of the different phases of migration. THNs expressed YFP-CAAX and are indicated by arrows and their migratory stage. Dotted line represents MHB, white line represents URL. Scale bar: 20 μm. (B) Hexamethonium does not change THN morphology, neither does the overexpression of GFP-AcheH (C). (D) Activating the glycine neurotransmitter system does not change THN morphology. (E) Neither CNQX nor (F) CNQX and hexamethonium treatment affect THN morphology (left and right panels). Adding NMDA (G), however, led to increased protrusions in ventrally located THNs (middle panel, compare to controls in Fig 4F). (H), (I) Magnification of THNs identified by “2” in (A) and (G) demonstrates the different morphologies in control (H) and excess NMDA (I) conditions Scale bar: 10 μm. CAAX, plasma membrane-targeting signal derived from K-Ras; CNQX, 6-cyano-7-nitroquinoxaline-2,3-dione; DMSO, dimethyl sulfoxide; MHB, midbrain-hindbrain boundary; NMDA, N-methyl-D-aspartate; THN, tegmental hindbrain nuclei neuron; URL, upper rhombic lip; YFP, yellow fluorescent protein.
(TIF)

S1 Data Calcium transients. All detected calcium transients are listed by their dataset identifiers.
(XLSX)

S2 Data THN tracking data—Optogenetics. (XLSX)

S3 Data THN tracking data—Drug screening. (XLSX)

S1 Table THN tracking statistics—Optogenetics. (XLSX)

S2 Table Drug screening assay conditions. (XLSX)

S3 Table THN tracking statistics—Drug screening. (XLSX)

S1 Video Overview of hindbrain development in zebrafish embryos. Main anatomical features are indicated in the first frame of the transmitted light images (left panel). THNs are labelled by GFP fluorescence in *atoh1a*:*KalTA/4xUAS-GFP*-positive embryos and migrate from the URL towards the MHB, which they follow ventrally to form clusters (right panel). Developmental stage is indicated as hpf. Scale bar: 100 μm. *atoh1a*, *atonal 1a*; GFP, green fluorescent protein; hpf, hours post fertilization; MHB, midbrain-hindbrain boundary; THN, tegmental hindbrain nuclei neuron; URL, upper rhombic lip.
(AVI)

S2 Video Morphology of THNs en route. Two embryos with THNs labelled by PM-targeted YFP are given (right panels). Transmitted light images outline the cerebellum (left panels). Arrows point to phase 1 migrating cells that retain their connection with the URL. Arrowheads indicate intermediate stage cells with many short-lived protrusions and stage 2 THNs with ventrally-oriented leading processes. Elapsed time is given in h:min. Scale bar: 20 μm. PM, plasma membrane; THN, tegmental hindbrain nuclei neuron; URL, upper rhombic lip; YFP, yellow fluorescent protein.
(AVI)

S3 Video Calcium signals are often recorded in front-oriented extensions. THNs in the cerebellum co-express GCamp6s and FyntagRFP-T to simultaneously visualize calcium (top panel) and the PM (lower panel). Ratiometric images show fluorescence of GCamp6s relative to the subsequent frame. Arrow indicates localized calcium signal. Elapsed time is given in min:s. Scale bar: 10 μm. GCamp6s, xxx; PM, plasma membrane; THN, tegmental hindbrain nuclei neuron.
(AVI)

S4 Video Optogenetic induction of de- or hyperpolarization produces opposite effects on THNs. Depolarization by imaging ChR2-expressing cells every minute leads to increased cell speed (second panel), which is not abolished by hexamethonium treatment (right panel). Hyperpolarization by opening SwiChR every 10 min results in a slowing down of THNs (third panel). THNs expressing optogenetic channels are identified by YFP. Individual cells are labelled with colored dots. Embryos were recorded in separate experiments. Elapsed time is given in h:min. Scale bar: 25 μm. ChR2, channelrhodopsin; SwiChR, mutated channelrhodopsin, THN, tegmental hindbrain nuclei neuron; YFP, yellow fluorescent protein.
(AVI)

S5 Video Track representation of THNs after optogenetic manipulation or optogenetic manipulation plus drug treatment and quantification. Several examples (orange) with one highlighted for reference (dark blue) are shown for one embryo after tissue shift correction, with correction markers represented in cyan. Embryos were recorded in separate experiments. Elapsed time is given in h:min. THN, tegmental hindbrain nuclei neuron.
(AVI)

S6 Video Long-term hyperpolarization delays THN progress and ventral cluster formation. Left panel: THNs expressing SwiChR without channel activation are shown in magenta. Right panel: THNs expressing SwiChR with blue light activation are shown in cyan. Transmitted light images are overlaid for reference. Embryos were recorded in separate experiments. Developmental stages are indicated as hpf. hpf, hours post fertilization; THN, tegmental hindbrain nuclei neuron; SwiChR, mutated channelrhodopsin.
(AVI)

S7 Video Hexamethonium treatment or overexpression of AcheH delays THN migration. Individual cells are labelled with colored dots. Embryos were recorded in separate experiments. Elapsed time is given in h:min. Scale bar: 25 μm. AcheH, Ache GPI-anchored isoform H; THN, tegmental hindbrain nuclei neuron.
(AVI)

S8 Video Track representation of THNs after hexamethonium or ACh treatment or AcheH overexpression and quantification. Several examples (orange) with one highlighted for reference (dark blue) are shown for one embryo after tissue shift correction, with correction markers represented in cyan. Embryos were recorded in separate experiments. Elapsed time is given in h:min. ACh, acetylcholine; AcheH, Ache GPI-anchored isoform H; THN, tegmental hindbrain nuclei neuron.
(AVI)

S9 Video Glycine-treatment or overexpression of GlyRa1 delays THN migration. Individual cells are labelled with colored dots. Embryos were recorded in separate experiments. Elapsed time is given in h:min. Scale bar: 25 μm. GlyRa1, glycine receptor alpha 1 subunit; THN, tegmental hindbrain nuclei neuron.
(AVI)

S10 Video Track representation of THNs after glycine treatment or glycine receptor subunit overexpression and quantification. Several examples (orange) with one highlighted for reference (dark blue) are shown for one embryo after tissue shift correction, with correction markers represented in cyan. Embryos were recorded in separate experiments. Elapsed time is given in h:min. THN, tegmental hindbrain nuclei neuron.
(AVI)

S11 Video Inhibition of glutamate perception by CNQX increases cell speed, while activation of the glutamate neurotransmitter system by NMDA reduces THN speed. Individual cells are labelled with colored dots. Elapsed time is given in h:min. Scale bar: 25 μm. CNQX, 6-cyano-7-nitroquinoxaline-2,3-dione; NMDA, N-methyl-D-aspartate; THN, tegmental hindbrain nuclei neuron.
(AVI)

S12 Video Track representation of THNs after CNQX or NMDA treatment and quantification. Several examples (orange) with one highlighted for reference (dark blue) are shown for one embryo after tissue shift correction, with correction markers represented in cyan. Embryos were recorded in separate experiments. Elapsed time is given in h:min. CNQX, 6-cyano-7-nitroquinoxaline-2,3-dione; NMDA, N-methyl-D-aspartate; THN, tegmental hindbrain nuclei neuron.
(AVI)

S13 Video Double treatment of THNs with CNQX and hexamethonium counterbalances the THN speed effects in the ventral portion of the MHB, but increases cell speed in the dorsal part. Individual cells are labelled with colored dots. Embryos were recorded in separate experiments. Elapsed time is given in h:min. Scale bar: 25 μm. CNQX, 6-cyano-7-nitroquinoxaline-2,3-dione; MHB, midbrain-hindbrain boundary; THN, tegmental hindbrain nuclei neuron.
(AVI)

S14 Video Track representation of THNs after CNQX, hexamethonium, or double treatment and quantification. Several examples (orange) with one highlighted for reference (dark blue) are shown for one embryo after tissue shift correction, with correction markers represented in cyan. Embryos were recorded in separate experiments. Elapsed time is given in h:min. CNQX, 6-cyano-7-nitroquinoxaline-2,3-dione; THN, tegmental hindbrain nuclei neuron.
(AVI)

S15 Video Long-term treatment of THNs with hexamethonium delays THN cluster formation, whereas CNQX treatment enhances cluster development. THNs in the cerebellum are visualized by the expression of GFP in *atoh1a*:*KalTA/4xUAS-GFP* embryos. Embryos were recorded in separate experiments. Developmental stages are indicated as hpf. Scale bar: 100 μm. *atoh1a*, *atonal 1a*; CNQX, 6-cyano-7-nitroquinoxaline-2,3-dione; GFP, green fluorescent protein; hpf, hours post fertilization; THN, tegmental hindbrain nuclei neuron.
(AVI)

## Abbreviations

ACh: acetylcholine
AchE: acetylcholinesterase
AcheH: Ache GPI-anchored isoform H
AMPA: α-amino-3-hydroxy-5-methyl-4-isoxazolepropionic acid receptor
*atoh1a*: *atonal 1a*
ChR2: channelrhodopsin
CMV: cytomegalovirus
CNQX: 6-cyano-7-nitroquinoxaline-2,3-dione
DMSO: dimethyl sulfoxide
F: fluorescence intensity
F/F0: fluorescence over background
F0: background intensity value
FFT: fast Fourier transform
GCaMP6: circular permutated green florescent protein-Calmodulin-M13 peptide 6
GFP: green fluorescent protein
GlyRa1: glycine receptor alpha1 subunit
GPI: glycosylphosphatidylinositol
H2B: Histone 2B
hpf: hours post fertilization
KCC2: solute carrier family 12 (potassium/chloride transporter), member 5b
MHB: midbrain-hindbrain boundary
MK801: dizocilpine
NMDA: N-methyl-D-aspartate
PM: plasma membrane
*ric3b*: RIC3 acetylcholine receptor chaperone b
ROI: region of interest
SwiChR: mutated channelrhodopsin
THN: tegmental hindbrain nuclei neuron
UAS: upstream activating sequence
URL: upper rhombic lip
WISH: whole mount in situ hybridization
wt: wild-type
Y226F: glycine receptor alpha1 subunit Y226 mutated to F
